# Diverse Phytochemicals and Bioactivities in the Ancient Fruit and Modern Functional Food Pomegranate (*Punica granatum*)

**DOI:** 10.3390/molecules22101606

**Published:** 2017-09-25

**Authors:** Sheng Wu, Li Tian

**Affiliations:** 1 Shanghai Key Laboratory of Plant Functional Genomics and Resources, Shanghai Chenshan Botanical Garden, Shanghai 201602, China; wusheng@mail.sioc.ac.cn; 2 Shanghai Chenshan Plant Science Research Center, Chinese Academy of Sciences, Shanghai 201602, China; 3 Department of Plant Sciences, University of California, Davis, CA 95616, USA

**Keywords:** anthocyanin, ellagic acid, ellagitannin, functional food, hydrolyzable tannin, polyphenol, pomegranate, *Punica granatum*, urolithin

## Abstract

Having served as a symbolic fruit since ancient times, pomegranate (*Punica granatum*) has also gained considerable recognition as a functional food in the modern era. A large body of literature has linked pomegranate polyphenols, particularly anthocyanins (ATs) and hydrolyzable tannins (HTs), to the health-promoting activities of pomegranate juice and fruit extracts. However, it remains unclear as to how, and to what extent, the numerous phytochemicals in pomegranate may interact and exert cooperative activities in humans. In this review, we examine the structural and analytical information of the diverse phytochemicals that have been identified in different pomegranate tissues, to establish a knowledge base for characterization of metabolite profiles, discovery of novel phytochemicals, and investigation of phytochemical interactions in pomegranate. We also assess recent findings on the function and molecular mechanism of ATs as well as urolithins, the intestinal microbial derivatives of pomegranate HTs, on human nutrition and health. A better understanding of the structural diversity of pomegranate phytochemicals as well as their bioconversions and bioactivities in humans will facilitate the interrogation of their synergistic/antagonistic interactions and accelerate their applications in dietary-based cancer chemoprevention and treatment in the future.

## 1. Introduction

Cultivation and consumption of pomegranate (*Punica granatum*) can be dated back to at least 3000 BC. Historically, pomegranate has served as a symbol of fertility and prosperity. In addition, various parts of the pomegranate have been used in traditional medicine for treating a wide variety of illness. Pomegranate fruits have purported use for expelling parasites [1], seeds and fruit peels for treating diarrhea [2,3], flowers for managing diabetes [4], tree barks and roots for stopping bleeding and healing ulcers [5], and leaves for controlling inflammation and treating digestive system disorders [5].

Due to its reported benefits to human health, the pomegranate has drawn great interest from the consumers in recent years. Nowadays, the pomegranate is used for functional food ingredients and dietary supplements in various forms, such as fresh fruit and juice, powdered capsules and tablets that contain extracts of different pomegranate tissues, tea brewed from pomegranate leaves, jam, jelly, juice and wine produced from pomegranate fruits, as well as spices prepared from dried seeds [6].

With the advancement of technologies and the expansion of experimental inquiries into the bioactivities of pomegranate phytochemicals, many new discoveries have been made in this ancient fruit within the last decade. To date, over 1500 articles have been published on the subject “pomegranate”, of which 1259 articles were published between 2006 and 2016. Although the pomegranate produces and accumulates a wide variety of phytochemicals with diverse structures in different tissues (Table 1), investigative efforts thus far have been given mainly to the bioactivities of polyphenols in pomegranate fruits, in particular anthocyanins (ATs) and hydrolyzable tannins (HTs), which are assessed in this review. Specifically, various health-promoting activities of urolithins, a group of phenolic metabolites transformed from ellagic acid (EA) (a hydrolysis product of pomegranate ellagitannins (ETs)) by the human gut microbiota, will be reviewed.

Development of cutting-edge analytical techniques has enabled the acquisition of large-scale metabolic datasets, which requires careful analysis and interpretation. To facilitate characterization of metabolite profiling data in pomegranate, we examine the phytochemicals that have been identified in pomegranate, including detailed information on the chemical structures, molecular formulas, molecular weights, analytical methods (reflecting confidence/accuracy of the identifications), and tissues of identification (Table 1 and Table S1). Knowledge of phytochemicals present in different pomegranate tissues will also help assess the structural determinants of their bioactivities as well as the additive, antagonistic or synergistic interactions of these phytochemicals in complex mixtures.

## 2. Occurrence and Structure of Diverse Phytochemicals in Pomegranate

Numerous phytochemicals have been (tentatively) identified in different pomegranate tissues using diode array detection (DAD), electron spin resonance (ESR), fluorescence detection (FD), flame ionization detection (FID), infrared spectroscopy (IR), mass spectrometry (MS), nuclear magnetic resonance (NMR), and thin layer chromatography (TLC; by comparing the R_f_ values of pomegranate phytochemicals to those of the authentic standards) (Table 1 and Table S1). It should be noted that disparities regarding the presence/absence of phytochemicals in specific tissues have been observed in different pomegranate cultivars [7,8,9,10]. In addition, the quantities of phytochemicals vary among the pomegranate cultivars [11].

### 2.1. Ellagitannins, Gallotannins, and Their Derivatives

HTs are among the most studied phytochemicals in pomegranate; they can be further grouped into ETs and gallotannins (GTs) based on the different phenolic acids that are esterified to the core cyclic polyol molecule (it is often a glucose molecule). Overall, more than 60 HTs have been identified from pomegranate using MS and/or NMR (Table 1). Pomegranate fruit peel is rich in HTs, particularly ETs. Punicalagin isomers (ETs) constitute up to 85% (*w*/*w*) of total tannins extracted from pomegranate fruit peel [12]. EA, methylated EA, and their glycosidic derivatives have also been found in fruit peel and other pomegranate tissues (Table 1). Although punicalagin isomers represent the major ETs in pomegranate roots, they accumulate at much lower levels in roots than fruit peel [13].

Besides fruit peel, pomegranate stem barks are also abundant in HTs and have been used historically in tanneries for making leather. In addition to the HTs identified in fruit peel, stem barks also contain ET *C*-glycosides, punicacorteins A–D (ETs with a gallagic acid component), and punigluconin (an ET with a gluconic acid core) [14]. The dense inner part of pomegranate tree trunk (i.e., heartwood) contains brevifolin carboxylic acid, EA rutinoside, diellagic acid rutinoside, methyl-EA, methyl-EA rutinoside, punicalin, galloylpunicalin, and galloylpunicacortein D [15,16,17].

The composition of HTs in pomegranate leaves is largely different from that in fruit peel. In leaves, the major HTs are granatins A and B, whereas punicalagins and punicalins are present at negligible levels [18]. Additional ETs with galloyl and/or hexahydroxydiphenoyl (HHDP) substitutions have also been identified in leaves [18,19,20]. Interestingly, derivatives of EA and ETs, including urolithin M-5, brevifolin, and brevifolin carboxylic acid, have been isolated from pomegranate leaves [19].

In pomegranate flowers, EA and two oxidized derivatives of EA, pomegranatate and phyllanthusiin E, were discovered [21]. Punicatannins A and B, two ETs that contain an unusual 3-oxol,3,3a,8b-tetrahydrofuro[3,4-b]benzofuran functional group, together with a structurally related compound isocorilagin, were also found in pomegranate flowers [22]. In addition, brevifolin carboxylic acid, ethylbrevifolin carboxylate, as well as glucose with various galloyl and/or HHDP substitutions, including hippomanin A, gemin D, digalloyl-diHHDP-glucose, trigalloyl glucose, and gallic acid 3-*O*-β-d-(6′-*O*-galloyl)-glucopyranoside showed measurable accumulations in pomegranate flowers [21,23,24].

### 2.2. Flavonoids

Pomegranate fruit peel, aril, and juice are abundant in flavonoids of diverse structures, including the aglycones and glycosides of chalcones, flavanones, flavones, flavonols, ATs, flavan-3-ols, and procyanidins (Table 1). Two flavones, luteolin and tricetin, were found in a methanolic extract of pomegranate flowers [23]. Structures of two flavanones, punicaflavanol (a misnomer; it is a flavanone) and granatumflavanyl xyloside, were elucidated by NMR [25], while hovetrichoside C (a glycoside of an auronol) and phlorizin (a glycoside of the dihydrochalone phloretin) were identified by IR in pomegranate flowers [24].

Similar to other plants, leaves of pomegranate also accumulate high levels of flavone (e.g., apigenin and luteolin) glycosides [19]. Two flavanone diglycosides and one flavonol diglycoside isolated from pomegranate stem barks were shown to be eriodictyol-7-*O*-α-l-arabinofuranosyl(1-6)-β-d-glucoside, naringenin-4′methyl ether 7-*O*-α-l-arabinofuranosyl(1-6)-β-d-glucoside, and quercetin-3,4′-dimethyl ether 7-*O*-α-l-arabinofuranosyl(1-6)-β-d-glucoside, respectively, by NMR analysis [26,27]. High performance liquid chromatography (HPLC)-DAD studies revealed that two isoflavones, genistein and daidzein, as well as a flavonol quercetin, are present in pomegranate seeds [9,28].

### 2.3. Lignans

Plant lignans are a group of phytoestrogens that can be metabolized into mammalian lignans by the gut microbiota. Furofuran-, dibenzylbutane-, and dibenzylbutyrolactone-type lignans have been identified in different pomegranate tissues based on liquid chromatography (LC)-MS^n^ studies (Table 1), while isolariciresinol is the most abundant lignan present in pomegranate fruit peel [29]. In addition to the above-mentioned lignans, pomegralignan, a dihydrobenzofuran-type neolignan glycoside, was discovered in the aril and fruit peel of pomegranate [30]. Another neolignan, punnicatannin C, was isolated from pomegranate flowers and structurally characterized by NMR analysis [24].

### 2.4. Triterpenoids and Phytosterols

Triterpenoids (C_30_) are the biosynthetic precursors of steroids in plants (phytosterols) and animals (steroid hormones). Triterpenoids and phytosterols have been found in pomegranate seed, leaf, flower, fruit peel, and bark tissues (Table 1). The presence of human steroid hormones, including estrone, estriol, estradiol, and testosterone, in pomegranate seeds was reported previously based on TLC separations and colorimetric assays [31,32]. However, HPLC-DAD- and gas chromatography (GC)-MS-based analysis showed that these steroid hormones could not be identified in pomegranate seeds using the more sensitive analytical methods [33].

### 2.5. Alkaloids and Indolamines

Pelletierine, pseudopelletierine, and *N*-methylpelletierine comprise the major alkaloids in pomegranate stem and root barks [34]. Sedridine, 2-(2′-hydroxypropyl)-∆^1^piperideine, 2-(2′-propenyl)-∆^1^piperideine, norpseudopelletierine, and the pyrrolidine alkaloids (with a five-membered *N*-containing ring) hygrine and norhygrine, were also found in root barks at low quantities [34]. In addition to the alkaloids that accumulate in root and stem barks, *N*-(2′,5′-dihydroxyphenyl)pyridinium chloride was identified in pomegranate leaves [19], and a pyrrolidine-type alkaloid punigratane (2,5-diheptyl-*N*-methylpyrrolidine) was recently characterized in pomegranate fruit peel [35]. Besides alkaloids, low levels of indolamines (amine derivatives of indole), including tryptamine, melatonin, and serotonin, were present in the extract of pomegranate fruit [36].

### 2.6. Fatty Acids and Lipids

Fatty acids (FAs) of medium (C_6_, C_8_, C_10_, and C_12_), long (C_14_, C_16_, C_18_, and C_20_), and very long (C_22_ and C_24_) chain length have been identified from pomegranate seeds, juice, and fruit using GC-FID, MS, or NMR analysis (Table 1). The polyunsaturated FA punicic acid (9*Z*, 11*E*, 13*Z*-octadecatrienoic acid) represents the most abundant FA in pomegranate seeds, accounting for over 60% of seed oil. Triacylglycerols (TAGs) containing 9*E*, 11*Z*, 13*E*-octadecatrienoic acid, 3-*O*-octadec-2-enoic acid, 9*Z*, 11*E*, 13*Z*-octadecatrienoic acid, and 8*Z*, 11*Z*, 13*E*-octadecatrienoic acid are produced in pomegranate seeds and their structures were determined by NMR [37,38,39]. In addition, a glycosphingolipid *N*-palmitoyl cerebroside was identified from pomegranate (cv. Nana) seed oil by TLC and GC-FID analyses [40].

### 2.7. Organic Acids and Phenolic Acids

The major organic acids in pomegranate juice are citric acid and malic acid [41]. Pomegranate juice also contains ascorbic acid, fumaric acid, oxalic acid, quinic acid, succinic acid, and tartaric acid, some of which have also been identified in the leaf, fruit peel, and seed tissues [8,9,41,42,43]. Phenolic acids (aromatic acids), primarily benzoic acid and cinnamic acid derivatives, are usually found in pomegranate fruit peel, juice, and flowers (Table 1). In addition, the structure of a substituted coumarin, 7,8-dihydroxy-3-carboxymethylcoumarin-5-carboxylic acid, was characterized in pomegranate flowers by NMR [24].

### 2.8. Other Compounds

Phenolic compounds that do not belong to HTs, flavonoids, and phenolic acids were also identified in pomegranate juice and seeds using DAD and MS (Table 1). These include catechol (a dihydroxybenzene), coumestrol (a coumestan), syringaldehyde (an aromatic aldehyde), as well as icariside D1 and phenylethylrutinoside (two phenylethanoid glycosides) [28,41,43,44].

## 3. Interactions of Pomegranate Phytochemicals

The synergy principle of phytochemicals has long been employed in traditional herbal medicine. Multi-target drugs derived from mixtures of plant natural products have increasingly been pursued nowadays to contradict drug tolerance and resistance in cancer therapy. Although several studies (discussed below) have suggested synergistic interactions among pomegranate phytochemicals, this is a promising, but currently under-explored topic.

Fresh arils and juice products of pomegranate fruit, as well as seeds of the soft-seeded cultivars, are mostly consumed. Phytochemicals in pomegranate fruit peel extracts, fermented pomegranate juice, and pomegranate seed oil exhibited cooperative interactions toward limiting the proliferation, metastasis, and invasiveness of human prostate cancer cells in vitro [45]. A subsequent analysis with pure phytochemicals, including EA, caffeic acid, luteolin, and punicic acid, also showed synergistic interactions on suppressing the invasion of prostate cancer cells [46]. Interestingly, commercial pomegranate juice demonstrated more antioxidant and anti-proliferative activities than the purified pomegranate polyphenols in colon cancer cells, suggesting that inherent synergies exist among polyphenols and other phytochemicals in pomegranate juice [47].

When the commercial pomegranate-nectarine juice was separated into predominately sugar, organic acid, neutral phenol, and AT fractions, complex antagonistic or synergistic effects were observed among different fractions on the total phenol or total antioxidant content [48]. The antagonistic or synergistic interactions depended on the concentrations of the chemical constituents in the juice product [49]. The polyphenol extracts of pomegranate fruit also synergistically interacted with the antibacterial drug ciprofloxacin, though various bacterial strains responded differently to the phytochemical-drug synergy, and the underlying mechanism of such synergy remains unknown [50].

## 4. Functions of Pomegranate ATs in Human Nutrition and Health

ATs are colorful, water-soluble polyphenol pigments that are found in many plant foods, such as berries and pomegranate fruits. Plant ATs are often investigated collectively as a group of phytochemicals for their bioactivities, and have been linked to many aspects of human disease prevention and treatment [51,52,53,54,55,56]. The anti-inflammatory and cardioprotective activities of ATs are attributed by their antioxidant properties via various underlying mechanisms [51]. ATs can quench free radicals, inhibit the activity of xanthine oxidase that generates free radicals, and chelate metal ions that are involved in oxidation of low-density lipoproteins (LDLs) [57]. In addition, ATs induce the expression of nuclear factor-erythroid 2-related factor-2 (Nrf2) that regulates the expression of endogenous antioxidant enzymes, such as hemeoxygenase-1 (HO-1) [51,55]. Besides reactive oxygen species (ROS), ATs also inhibit the production of reactive nitrogen species (RNS), particularly nitric oxide (NO), as well as their associated oxidative processes [58]. Furthermore, release of pro-inflammatory mediators and adhesion molecules is suppressed by ATs via targeting of the respective signaling pathways, e.g., the arachidonic acid and the tumor necrosis factor (TNF)-α, nuclear factor (NF)-κB pathways [59,60].

Aside from cardioprotection, the anti-inflammatory property of ATs also contributes to their anti-obesity effect and action in adipose tissue [53]. Studies using AT-rich fruit extracts showed that ATs prevented the upregulation of inflammatory response in adipose tissue, when triggered with consumption of a high-fat diet, by regulating the NF-κB stress signaling pathway [61]. ATs also reduce the level of pro-inflammatory cytokines (mediators of cell-to-cell communications in immune response) [62] and modulate the expression of adipocytokines (mediators linking adipose tissue, inflammation and immunity) [62,63]. Although studies using cell lines and animal models support the anti-obesity effect of ATs, a causal relationship between AT consumption and reduction of body mass index has yet to be conclusively established by clinical studies [64]. This could be due to one or more parameters that are associated with different clinical studies, such as the amount and types of ATs ingested, the food matrix of ATs, and the inherent difference of the study subjects (e.g., different age groups of the participants) [53].

In addition to their antioxidant and anti-inflammatory activities, ATs act in cancer chemoprevention by inducing terminal differentiation of tumor cells and thus impeding tumorigenesis [65]. Prevention of malignant cell transformation and inhibition of cancer cell proliferation by ATs have also been reported [66]. ATs can further interfere with cancer development by activating caspases and inducing apoptosis of cancer cells [67]. However, the cancer chemotherapeutic effect of ATs still requires strong supporting evidence from clinical studies. In addition, the bioconversion of ATs by human intestinal microbiome should be rigorously investigated.

Similar to other AT-rich fruits, the pomegranate also has a cardioprotective effect by targeting two major causal factors of atherosclerotic lesion and cardiovascular disease, namely the accumulation of cholesterol and oxidized lipids, and arterial macrophage foam cell formation [6]. Pomegranate juice and fruit extracts are also associated with antidiabetic activities, largely due to the antioxidant properties of HTs and ATs in reducing oxidative stress and lipid peroxidation [4]. Supplements of pomegranate juice or fruit extracts (including juice, fruit peel and seeds) at 1 g/kg/day for five weeks, effectively enhanced endothelial NO synthase (eNOS) expression, as well as plasma nitrate and nitrite (end products of NO) levels in plasma in obese Zucker rats fed with an atherogenic diet [68]. In addition, supplements of juice (extracted from pomegranate arils) at 100 mg/kg/day or 300 mg/kg/day for four weeks prevented the development of high blood pressure in diabetic rats [69]. Consumption of pomegranate juice rich in ATs and HTs also lowered systolic and diastolic blood pressure in hypertensive patients [70].

The bioactivities of pomegranate ATs have mostly been studied in the context of pomegranate juice and fruit extracts. However, these AT-rich sources are abundant in vitamin C, carotenoids, and HTs, which can also contribute to the ascribed bioactivities. To this end, “white” (AT-less) pomegranate cultivars are available, which lack the accumulation of ATs in leaves, fruits (including peels and arils) and flowers [71,72]. Comparative analysis of the white and AT-rich pomegranate cultivars will be informative as to the specific role of ATs in the health-promoting activities of pomegranate juice and fruit extracts.

## 5. Roles of Urolithins, the ET-Derived Metabolites, in Human Nutrition and Health

ETs are abundant in pomegranate fruit peel, as well as juice and extracts that are produced commercially from the whole fruit. Besides pomegranate, ETs are also present in a wide range of medicinal and food plants, such as many berries, nuts, and herbs [73,74]. As such, the knowledge of biotransformation and bioconversion of pomegranate ETs by human and microbial enzymes (discussed in this section) will inform nutritional and pharmacological studies on ETs that are isolated from other plants.

**Figure 1:**
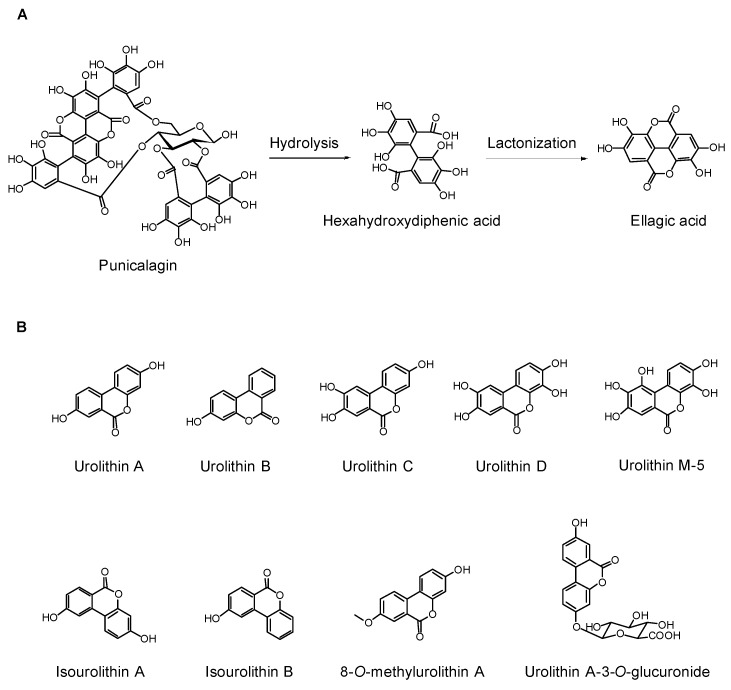
Pomegranate ellagitannin-derived metabolites. (**A**) Punicalagin (a pomegranate ellagitannin) is hydrolyzed to hexahydroxydiphenic acid, which is lactonized to yield ellagic acid; (**B**) Chemical structures of representative urolithins, the microfloral transformation products of ellagic acid.

EA, a hydrolysis product of ETs (Figure 1A), can be detected (at a maximum concentration of 0.06 μM) in the blood circulation of healthy volunteers within half an hour of ingesting pomegranate juice or extracts [75]. The human gut microbiota can further convert EA into urolithins prior to their absorption by the intestinal cells. It was shown that urolithin concentrations reached up to 18.6 μM in the plasma of healthy volunteers after consuming pomegranate juice for five consecutive days [76].

Urolithins contain a common core structure of dibenzopyranone and are more hydrophobic than EA (Figure 1B). The microfloral transformation of EA to urolithins likely involves cleavage of the lactone ring of EA by an esterase, followed by removal of the carboxyl group resulted from the ring opening by a decarboxylase, and then removal of one or more hydroxyl groups by an oxidoreductase [77]. Urolithin A (UA), urolithin B (UB), hydroxyl-UA, UA-glucuronide, and dimethyl EA-glucuronide have been identified in the plasma of healthy individuals after taking pomegranate extracts [78]. Additional urolithins, such as isourolithin A (isoUA), have also been reported in human urine or stool samples [79]. Emerging experimental evidence has suggested that urolithins play a major role in the anti-cancer, anti-inflammatory, and anti-aging activities of pomegranate fruit and products, which are discussed in the following sections.

### 5.1. Breast and Endometrial Cancers

Aromatase that converts androgens to estrogens has been considered to be a therapeutic target for treating the hormone-sensitive type of breast cancer [80]. Although several urolithins (at 47 μM) exhibited anti-aromatase activities in a placental microsome-based enzyme assay, only UB competitively inhibited and most effectively suppressed the aromatase activity in an aromatase-overexpressing breast cancer cell line (MCF-7aro) [81]. Consistent with “in cell” aromatase-inhibitory activity, UB also significantly arrested testosterone-induced proliferation of MCF-7aro cells, suggesting an underlying mechanism of aromatase inhibition. Interestingly, estrogen-induced proliferation of MCF-7aro cells was also inhibited by urolithins. However, inhibition was was likely effected through mechanisms independent of the aromatase activity [81].

Besides breast cancer, urolithins can also reduce the risks of another sex steroid hormone-related cancer, endometrial cancer. UA and UB (at 10 μM for 48 h) caused over 50% reduction in the proliferation of human endometrial cancer cells [82]. UA (at 10 μM and 50 μM for 48 h) arrested cell cycle at the G2/M phase and regulated the expression of cell cycle-related proteins at this phase. On the other hand, UA was shown to act as an estrogen agonist and modulate the estrogen-receptor α (ERα)-dependent gene expression in the ER-positive endometrial cancer cells. It remains to be determined whether and how estrogen signaling could be involved in the suppression of endometrial cancer cell growth by urolithins [82].

### 5.2. Prostate Cancer

UA (IC_50_ = 35.2 ± 3.7 μM), UB (IC_50_ > 40 μM), and urolithin C (UC; IC_50_ = 46.5 ± 1.6 μM) impaired the proliferation of the “androgen-sensitive” prostate cancer cells (LNCaP) [83]. UA and UC also attenuated the release of the prostate specific antigen (PSA) by the cancer cells, and inhibited the activities of arginase (a hydrolase that converts arginine to ornithine and urea and is critical for the proliferation of prostate cancer cells) [83]. Further investigations of the regulatory mechanism of urolithins indicated that UA and UB repressed the transcription and translation of the androgen receptor (AR), and subsequently reduced PSA transcripts and proteins in the LNCaP cells [84]. Urolithins also induced apoptosis in the LNCaP cells, which correlated with a decrease in the anti-apoptotic B-cell lymphoma 2 (Bcl-2) proteins, an increase in the apoptotic-protective Cyclin Dependent Kinase Inhibitor 1A (CDKN1A) transcripts and proteins, and an activation of the apoptotic cysteine proteases caspases 3 and 7 [84,85].

Cytochrome P450 1 (CYP1) family members activate pro-carcinogens and often exhibit enhanced expression in tumor and cancer cells [86]. UA (IC_50_ = 1.15 ± 0.65 μM), UB (IC_50_ = 1.55 ± 0.49 μM), and methyl-UA (mUA; IC_50_ = 1.49 ± 0.39 μM) effectively inhibited the CYP1-mediated ethoxy resorufin-*O*-deethylase (EROD) activities and were selective toward CYP1-B1 over CYP1-A1 (CYP1-B1 and CYP1-A1 are two members of the CYP1 family) in in vitro assays [87]. Incubation with UA (IC_50_ = 32 ± 8.9 μM) and UB (IC_50_ = 38 ± 3.9 μM) also led to inhibition of the 2,3,7,8-tetrachlorodibenzo-*p*-dioxin (TCDD)-induced CYP1 activities and decreased CYP1-B1 protein accumulation in the “androgen-responsive” 22Rv1 prostate cancer cells. However, the anti-proliferative effects of UC, dimethyl-UC, and mUA toward 22Rv1 cells were due to their cytotoxic activities rather than inhibition of the TCDD-induced CYP1 expression [87].

In the “androgen-independent” DU145 and PC3 prostate cancer cell lines, EA and UA synergistically inhibited cancer cell proliferation [88]. However, EA and UA exerted differential impacts on cell cycle control and induction of apoptosis in DU145 and PC3 cancer cells, suggesting that different molecular targets may exist for these pomegranate-derived metabolites [88]. Caspase-dependent cell apoptosis was induced in DU145 cells by mUA treatment [89]. Upon exposure to mUA (at 40 μM or 60 μM), expression of the oncogenic microRNA-21 (miR-21) was reduced, whereas protein levels of the miR-21 targets, including phosphatase and tensin homolog (PTEN), programmed cell death 4 (Pdcd4), and forkhead box O3a (FOXO3a), were elevated. In addition, the expression of protein kinase B (Akt), as well as β-catenin and its downstream oncogenic transcription factors, was decreased by the mUA treatment [89]. These data suggest that the mUA suppression of DU145 cell growth is mediated by regulation of miR-21 expression and the downstream PTEN/Akt and Wnt/β-catenin signaling pathways.

The Eph receptor-Ephrin ligand signaling system is implicated in an array of cellular processes, such as cancer development. UC and urolithin D (UD) reduced specifically the EphA2-Ephrin-A1 interaction (this receptor-ligand pair is known to be involved in cancer development and progression) of the Eph-Ephrin system [90]. UD acts as a protein-protein antagonist of EphA2 (a receptor kinase) by interfering its phosphorylation mediated by Ephrin-A1, without inhibiting the kinase domain of EphA2, and using a mechanism other than cytotoxicity or anti-proliferative effect in the PC3 human prostate adenocarcinoma cells [90].

Overall, these studies collectively suggest that urolithins may find applications as chemo-preventive agents against prostate cancers, through different mechanisms in androgen-sensitive, -responsive, and -independent cells.

### 5.3. Colon and Bladder Cancers

The canonical Wnt/β-catenin signaling pathway has been shown to activate T-cell factor transcription and function in colon carcinogenesis [91]. To determine the potential involvement of urolithins in colon carcinogenesis through Wnt signaling, phenolic extracts of several fruits rich in ETs, including pomegranate, strawberry (*Fragaria ananassa*), and Jamun berry (*Eugenia jambolana*), as well as pure EA and UA compounds, were tested in the human embryonic kidney 293T cells that express a reconstructed canonical Wnt signaling pathway [92]. Although the fruit extracts and chemicals all inhibited Wnt signaling, only UA exhibited an IC_50_ value (at 39 μM) that was physiologically relevant in the lumen of colons when taking enterohepatic circulation of urolithins into consideration [92].

During the initiation stage of the HT-29 colon cancer cells, urolithins A–D inhibited TCDD-induced, CYP1-mediated EROD activities [93]. At the cancer progression stage, UA and UB (at 30 μg mL^−1^ for 48 h) impaired the proliferation of HT-29 cells and led to cell cycle arrest at the G2/M phase, as well as activation of *CDKN1A* expression. In addition, treatment with urolithins resulted in activation of caspases 3, 8, and 9, suggesting that urolithins induced both the extrinsic (death receptor-mediated; where caspase 8 is activated) and intrinsic (mitochondrial mediated; where caspase 9 is activated) apoptotic pathways in HT-29 cells [94].

UA, UB, and mUA inhibited the proliferation of the T24 human bladder cancer cells in vitro, with IC_50_ values of 43.9, 35.2, and 46.3 μM, respectively [10], comparable to the UA inhibition of Wnt signaling at an IC_50_ of 39 μM [92]. The transcript and protein levels of Phospho-p38 mitogen-activated protein kinase (MAP kinase; MAPK) were increased by the urolithin treatment, while those of MAP kinase kinase kinase1 (MEKK1) and Phospho-c-Jun were decreased in the T24 cells. Furthermore, these urolithins reduced the level of H_2_O_2_-induced oxidative stress and induced apoptosis through activation of caspase 3 and PPAR-γ protein expression [10].

### 5.4. Cardiovascular Diseases

Inflammatory responses involving the activation of neutrophils and monocytes play a central role in the development of cardiovascular diseases [95]. Intriguingly, the number of free hydroxyl functional groups on urolithins appeared to impact how they modulate the inflammatory functions of neutrophils [96]. Among the urolithins tested (including UA, UB, UC, mUA, and methyl-urolithin C/mUC), UA (with two free hydroxyl groups; at 1 μM) exhibited the most potent antioxidant activities against the release of ROS from the pro-inflammatory triggered neutrophils. UB (with one free hydroxyl group; at 20 μM) significantly affected several inflammatory biomarkers that are associated with cardiovascular events, by inhibiting the production of interleukin 8 (IL8) and metalloproteinase-9 (MMP-9), and preventing the shedding of selectin CD62L triggered by the pro-inflammatory factor cytochalasin A/formyl-met-leu-phenylalanine (f-MLP). UC (with three free hydroxyl groups; at 5 μM), on the other hand, inhibited the release of elastase, a pro-inflammatory mediator responsible for extracellular matrix (ECM) degradation, from the f-MLP-stimulated neutrophils [96].

In addition to recruitment of neutrophils, monocyte adhesion to endothelial cells represents another key event in inflammatory responses [95]. A mixture of UA and UB (each at 10 μM) restricted the adhesion of Tamm-Horsfall protein-1 (THP-1) monocytes to the human umbilical vein endothelial cells (HUVECs) [97]. UA glucuronide (at 15 μM), but not UA, UB or UB glucuronide, inhibited monocyte adhesion to TNFα-stimulated human aortic endothelial cells (HAECs); a dosage-dependent inhibition of TNFα-induced migration of endothelial cells was also shown for the above-mentioned urolithins [98].

NO plays multifaceted roles in combatting cardiovascular diseases [99]. Although UA, UB, and UB glucuronide did not show any effect individually at 15 μM on NO bioavailability, a mixture of the three urolithins at equal concentrations (total 15 μM) activated the expression of eNOS after a 5-min incubation and increased NO production in primary HAECs after a 24-h incubation [100]. Overall, these in vitro studies with neutrophils, monocytes, and NO suggested the potential anti-inflammatory and cardiovascular-protective functions of urolithins.

### 5.5. Obesity

Based on the urinary excretion of urolithins by healthy volunteers after ingesting ET-rich foods or fruit extracts, three urolithin metabolic types (metabotypes) have been defined, including A (excretes only UA), B (excretes UA, UB, and isoUA), and 0 (does not excrete urolithins) [79]. Interestingly, the population of the human gut bacteria *Gordonibacter urolithinfaciens* correlated positively with the in vivo production of UA, but inversely with that of UB and isoUA [101]. A recent study observed an interlinked relationship among gut dysbiosis (i.e., microbial imbalance), ET metabolism, and obesity [102]. A relatively high percentage of metabotype B was found in the overweight-obese group, while metabotype A had a higher presentation in the normoweight (i.e., normal weight) than the overweight-obese group. In addition, *G. urolithinfaciens* levels were higher in the metabotype A than the metabotype B individuals [102]. Further investigations should provide a mechanistic understanding of how consuming the polyphenol precursors of urolithins, in the presence of UA-producing bacteria, may reduce the risks of diseases associated with obesity.

Large inter-individual variations in the cardiovascular risk biomarkers were observed in healthy overweight-obese individuals after consuming pomegranate supplement [103]. However, after clustering the different urolithin metabotypes in these individuals, improved blood lipid profiles were evident in the metabotype B group, in a dose-dependent fashion, while there was no significant effect in the metabotype A group. Interestingly, several metabotype 0 (urolithin non-producers; according to baseline analysis) individuals shifted to metabotype A or B (urolithin producers) after consuming pomegranate extracts [103]. Together with the study by Selma et al. (2016), these results suggest that consumption of pomegranate extracts may have personalized effects that are associated with the gut microbiota and the urolithin metabotypes of the individuals.

### 5.6. Aging

Of the various pomegranate phenolic metabolites exhibiting anti-Alzheimer activities in in vitro assays, only urolithins, including UA, UB, mUA, and methyl-urolithin B (mUB), were predicted to be capable of crossing the blood-brain barrier [104]. Methylation of urolithins by the mammalian enzymes may further improve their lipophilicity and facilitate the penetration of the blood-brain barrier. Corroborating with the computational predictions, urolithins reduced the production of the neurotoxic, fibrillogenic β-amyloid (Aβ) peptide in vitro. Significant improvement of the survival/mobility of *Caenorhabditis elegans* (post-induction by Aβ_1-42_ for muscular paralysis) was also achieved by mUB treatment [104]. However, empirical evidence is still needed to verify the roles of urolithins in preventing Alzheimer’s disease (age-related memory loss) in vivo.

Advanced protein glycation (non-enzymatic glycosylation) products have been implicated in the development of chronic diseases, such as diabetes and Alzheimer’s disease. The inhibitory effects of pomegranate fruit extract, punicalagin, EA, gallic acid (GA), UA, and UB toward the early, middle, and late stage of protein glycation were compared using in vitro assays [105]. Although all phenolic metabolites showed anti-glycation activities, pomegranate extract, punicalagin, and EA were more effective than GA, UA, and UB in preventing the glycation of proteins by fructose [105].

The mechanistic basis underlying UA’s anti-aging activities was explored recently using *C. elegans*, mammalian cells, and rodent models [106]. Feeding *C. elegans* with UA led to a dosage-dependent extension of the worm’s lifespan. It was shown that UA could activate mitophagy (degradation of mitochondria by autophagy) and eliminate damaged mitochondria in *C. elegans*, mammalian cells derived from muscle or intestinal tissues, and muscles of young and old rodents. UA was also found to be bioavailable in the skeletal muscle and could improve muscle function in mice and rats [106]. The role of urolithins in enhancing mitochondrial function and muscle quality presents promising dietary leads (e.g., ET-rich foods) for improved mobility in the elderly population.

## 6. Future Perspectives

Pomegranate fruits, leaves, flowers, and seeds have all been used in traditional herbal medicine for treating various illness. However, bioactive metabolites present in pomegranate fruit peel and juice have received considerable more attention than those found in other tissues. Bioactivities of the phytochemicals accumulating in tissues other than fruit should be investigated more rigorously in the future. In addition, detailed metabolite profiling and characterization of different pomegranate cultivars, grown under non-standard conditions (e.g., subjected to biotic and abiotic stresses), can be carried out to explore further the phytochemical diversity in pomegranate. 

Metabolic enzymes and pathways that confer the structural diversity of pomegranate phytochemicals also warrant further investigation. For example, the production of diverse HT monomers and polymers has been suggested to involve the activity of multiple laccase enzymes. However, the HT-forming laccases have not been cloned and characterized from pomegranate or any other plant species. Elucidation of the laccase family enzymes in pomegranate that bring about the great diversity of HT structures will have broad implications in dissecting the biological functions of HTs.

In vitro assays and animal model studies have demonstrated various health benefits of urolithins, the ET derivatives. It is critical to obtain clinically relevant data on their efficacies prior to widespread applications in human disease interventions. Overall, exploration of the diversity and interactions of pomegranate phytochemicals, as well as preclinical and clinical investigations of their bioactivities, holds great promise of fully realizing the potential of this ancient fruit and modern functional food. Since a majority of the phytochemicals identified in pomegranate have also been found in other plants, examining the interactions and health-promoting functions of pomegranate phytochemicals will also have a far-reaching impact on exploiting the bioactive components of various edible medicinal plants and functional plant food.

## Figures and Tables

**Table 1:** Different classes of phytochemicals identified from pomegranate. Detailed descriptions of the chemical structures, molecular formulas, molecular weights, tissues of identification, and representative references are presented in Supplementary Table S1.

Classes	Phytochemicals
Ellagitannins, gallotannins and derivatives	Brevifolin, Brevifolin carboxylic acid, Brevifolin carboxylic acid 10-monopotassium sulphate, Castalagin, Casuariin, Casuarinin, Corilagin, Isocorilagin, Hippomanin A, Gemin D, Diellagic acid rhamnosyl(1→4) glucopyranoside, 1,2-Di-*O*-galloyl-4,6-*O*-(*S*)-hexahydroxydiphenoyl β-d-glucopyranoside, Ellagic acid, 3,3′-Di-*O*-methylellagic acid, 3,3′,4′-Tri-*O-*methylellagic acid, 3-*O-*Methylellagic acid, 4,4′-Di-*O*-methylellagic acid, 3′-*O-*Methyl-3,4-methylenedioxy-ellagic acid, Eschweilenol C (Ellagic acid 4-*O*-α-l-rhamnopyranoside), Ethyl brevifolincarboxylate, Eucalbanin B, Eucarpanin T_1_, Pomegraniin A, Pomegraniin B, Gallagic acid, Gallic acid 3-*O*-β-d-(6′-*O*-galloyl)-glucopyranoside, 6-*O*-Galloyl-2,3-(*S*)-hexahydroxydiphenoyl-d-glucose, 5-Galloylpunicacortein D, 2-*O*-Galloylpunicalin (2-*O*-Galloyl-4,6-(*S*,*S*)-gallagyl-d-glucose), Granatin A, Granatin B, 2,3-(*S*)-Hexahydroxydiphenoyl-d-glucose, Lagerstannin B, Lagerstannin C, 3-*O*-Methylellagic acid 4-*O*-α-l-rhamnopyranoside, 3,4′-*O*-Dimethylellagic acid 4-*O*-α-l-rhamnopyranoside, Oenothein B, Pedunculagin I, Pedunculagin II, 1,2,3,4,6-Penta-*O*-galloyl-β-d-glucose, 3,4,8,9,10-Pentahydroxydibenzo [*b,d*] pyran-6-one (Urolithin M-5), Phyllanthusiin E, Pomegranatate, Punicacortein A, Punicacortein B, Punicacortein C, Punicacortein D, Punicafolin, Punicalagin A, Punicalagin B, Punicalin, Punicatannin A, Punicatannin B, Punigluconin, Strictinin [1-*O*-Galloyl-4,6-(*S*)-hexahydroxydiphenoyl-d-glucose], Tellimagrandin I, Tercatain [1,4-Di-*O*-galloyl-3,6-(*R*)-hexahydroxydiphenoyl-β-glucopyranose], Terminalin (Gallagyl dilactone), 1,2,4,6-Tetra-*O*-galloyl-β-d-glucose, 1,2,3-Tri-*O*-galloyl-β-glucopyranose, 1,2,4-Tri-*O*-galloyl-β-glucopyranose, 1,2,6-Tri-*O*-galloyl-β-glucopyranose, 1,3,4-Tri-*O*-galloyl-β-glucopyranose, 1,4,6-Tri-*O*-galloyl-β-glucopyranose, 3,4,6-Tri-*O*-galloyl-β-glucopyranose, Valoneic acid dilactone
Flavonoids	Hovetrichoside C, Phloretin, Phlorizin, Eriodictyol-7-*O*-α-l-arabinofuranosyl (1-6)-β-d-glucoside, Granatumflavanyl xyloside, Naringin (Naringenin-7-*O*-rhamnoglucoside), Naringenin-4′methyl ether 7-*O*-α-l-arabinofuranosyl(1-6)-β-d-glucoside, Pinocembrin, Punicaflavanol, Apigenin, Apigenin 4′-*O*-β-glucopyranoside, Luteolin, Luteolin 3′-*O*-β-glucopyranoside, Luteolin 4′-*O*-β-glucopyranoside, Cynaroside (Luteolin 7-*O*-glycoside), Luteolin 3′-*O*-β-xylopyranoside, Tricetin, Daidzein, Genistein, Amurensin (Noricaritin 7-β-d-glucopyranoside), Kaempferol, Astragalin (Kaempferol 3-*O*-glucoside), Kaempferol-3-*O*-rhamnoglucoside, Myricetin, Phellatin, Quercetin, Hirsutrin (Quercetin-3-*O*-glucoside), Quercimeritrin (Quercetin-7-*O*-glucoside), Quercetin 3-*O*-rhamnoside, Rutin (Quercetin-3-*O*-rutinoside), Quercetin-3,4′-dimethyl ether 7-*O*-α-l-arabinofuranosyl(1-6)-β-d-glucoside, Cyanidin, Chrysanthemin (Cyanidin-3-*O*-glucoside), Cyanin (Cyanidin-3,5-di-*O*-glucoside), Antirrhinin (Cyanidin-3-*O*-rutinoside), Catechin-cyanidin-3-hexoside, Delphinidin, Myrtillin (Delphinidin-3-*O*-glucoside), Delphinidin-3,5-di-*O*-glucoside, Pelargonidin, Callistephin (Pelargonidin-3-*O*-glucoside), Pelargonin (Pelargonidin-3,5-di-*O*-glucoside), Catechin, Epicatechin, Epicatechin gallate, Epigallocatechin-3-*O*-gallate, Gallocatechin-(4→8)-catechin, Gallocatechin-(4→8)-gallocatechin, Catechin-(4→8)-gallocatechin, Procyanidin A2, Procyanidin B1, Procyanidin B2, Procyanidin B3
Lignans	Conidendrin, Isohydroxymatairesinol, Isolariciresinol, Matairesinol, Medioresinol, Phylligenin, Pinoresinol, Secoisolariciresinol, Syringaresinol, Pomegralignan, Punicatannin C
Triterpenoids and phytosterols	Asiatic acid, Betulinic acid (Betulic acid), Friedooleanan-3-one (Friedelin), Maslinic acid, Oleanolic acid, Punicanolic acid, Ursolic acid, Campesterol, Cholesterol, Daucosterol, β-Sitosterol, β-Sitosterol laurate, β-Sitosterol myristate, Stigmasterol
Alkaloids and indolamines	*N*-(2′,5′-Dihydroxyphenyl)pyridinium chloride, Hygrine, Norhygrine, Pelletierine, *N*-Methylpelletierine, Norpseudopelletierine, Pseudopelletierine, 2-(2′-Hydroxypropyl)-∆^1^piperideine, 2-(2′-Propenyl)-∆^1^piperideine, Punigratane (2,5-Diheptyl-*N*-methylpyrrolidine), Sedridine, Melatonin, Serotonin, Tryptamine
Fatty acids and lipids	Caproic acid (Hexanoic acid), Caprylic acid (Octanoic acid), Capric acid (Decanoic acid), Lauric acid (Dodecanoic acid), Myristic acid (Tetradecanoic acid), Myristoleic acid (9-*cis*-Tetradecanoic acid), Palmitic acid (Hexadecanoic acid), Palmitoleic acid (Hexadec-9-enoic acid), Punicic acid (9*Z*, 11*E*, 13*Z*-octadecatrienoic acid), Linoleic acid (*cis*, *cis*-9,12-Octadecadienoic acid), α-Linolenic acid (All-*cis*-9,12,15-octadecatrienoic acid), γ-Linolenic acid (All-*cis*-6,9,12-octadecatrienoic acid), Oleic acid (9*Z-*octadecenoic acid), Stearic acid (Octadecanoic acid), α-Eleostearic acid (9*Z*, 11*E*, 13*E*-octadecatrienoic acid), β-Eleostearic acid (9*E*, 11*E*, 13*E*-octadecatrienoic acid), Catalpic acid (9*E*, 11*E*, 13*Z*-octadecatrienoic acid), Arachidic acid (Eicosanoic acid), Gadoleic acid (9*Z*-icosenoic acid), Behenic acid (Docosanoic acid), Nervonic acid (*cis*-15-Tetracosenoic acid), 1-*O*-9*E*,11*Z*,13*E*-Octadecatrienoyl glycerol, 1-*O*-Isopentyl-3-*O*-octadec-2-enoyl glycerol, Tri-*O*-punicylglycerol, Di-*O*-punicyl-*O*-octadeca-8*Z*, 11*Z*, 13*E*-trienylglycerol, *N*-palmitoyl cerebroside
Organic acids and phenolic acids	Ascorbic acid, Citric acid, Fumaric acid, l-Malic acid, Oxalic acid, Quinic acid, Succinic acid, Tartaric acid, Caffeic acid, Chlorogenic acid, Cinnamic acid, *o*-Coumaric acid, *p*-Coumaric acid, *cis-p*-Coumaric acid, Coutaric acid, 7,8-Dihydroxy-3-carboxymethylcoumarin-5-carboxylic acid, Ferulic acid, Gallic acid, Methyl gallate, Neochlorogenic acid (5-*O*-Caffeoylquinic acid), Protocatechuic acid, Vanillic acid, Coniferyl 9-*O*-[*β*-d-apiofuranosyl(1→6)]-*O*-*β*-d-glucopyranoside, Sinapyl 9-*O*-[*β*-d-apiofuranosyl(1→6)]-*O*-*β*-d-glucopyranoside
Other compounds	Catechol, Coumestrol, Icariside D1, Phenylethylrutinoside, Syringaldehyde

## Supplementary Materials

The following are available online, Table S1: Phytochemicals identified from different pomegranate tissues.
