# Supplementary material for: Diverse Phytochemicals and Bioactivities in the Ancient Fruit and Modern Functional Food Pomegranate (Punica granatum)
Source: Molecules. 2017 Sep 25;22(10):1606. doi: 10.3390/molecules22101606 (PMC6151597; doi:10.3390/molecules22101606)
Supplement: Supplementary file 1 [file molecules-22-01606-s001.pdf]

molecules

**Table S1.** Phytochemicals identified from different pomegranate tissues. The chemical structures, molecular formulas, and molecular weights of the phytochemicals are shown. The analytical methods, tissues of identification, and representative references are also indicated. CAS, chemical abstracts service; DAD, diode array detection; ESR, electron spin resonance; FD, fluorescence detection; FID, flame ionization detection; ID, identification method; IR, infrared spectroscopy; MP, melting point; MS, mass spectrometry; MW, molecular weight; NMR, nuclear magnetic resonance; TLC, thin layer chromatography.

| Name | Structure | Formula | MW | ID | Tissue | References |
|---|---|---|---|---|---|---|
| **Ellagitannins, gallotannins and derivatives** | | | | | | |
| Brevifolin | 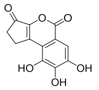 | $C_{12}H_8O_6$ | 248.1900 | NMR | Leaf | [1] |
| Brevifolin carboxylic acid | 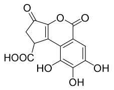 | $C_{13}H_8O_8$ | 292.1990 | NMR | Leaf, flower, heartwood | [1-3] |
| Brevifolin carboxylic acid 10-monopotassium sulphate | 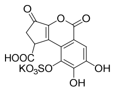 | $C_{13}H_7KO_{11}S$ | 410.3463 | NMR | Leaf | [4] |
| Castalagin | 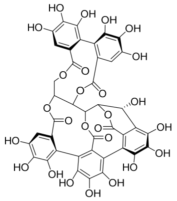 | $C_{41}H_{26}O_{26}$ | 934.6330 | NMR | Stem bark | [5] |
| Casuariin | 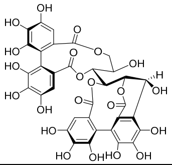 | $C_{34}H_{24}O_{22}$ | 784.5440 | NMR | Stem bark | [5] |
| Casuarinin | 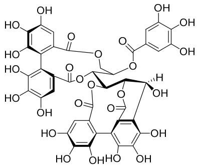 | $C_{41}H_{28}O_{26}$ | 936.6490 | NMR | Peel, stem bark | [5, 6] |
| | 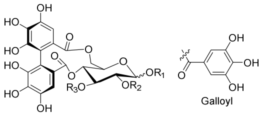 | | | | | |
| Corilagin | $R_1$ = Galloyl (β-configuration), $R_2 = R_3$ = H | $C_{27}H_{22}O_{18}$ | 634.4550 | NMR | Peel, leaf | [6, 7] |
| Isocorilagin | $R_1$ = Galloyl (α-configuration), $R_2 = R_3$ = H | $C_{27}H_{22}O_{18}$ | 634.4550 | NMR | Flower | [8] |
| Hippomanin A | $R_2$ = Galloyl, $R_1 = R_3$ = H | $C_{27}H_{22}O_{18}$ | 634.4550 | IR | Flower | [2] |
| Gemin D | $R_1 = R_2$ = H, $R_3$ = Galloyl | $C_{27}H_{22}O_{18}$ | 634.4550 | IR | Flower | [2] |
| Diellagic acid rhamnosyl(1→4) glucopyranoside | 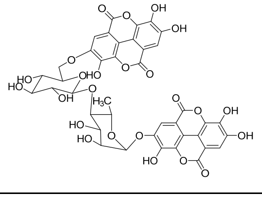 | $C_{40}H_{30}O_{24}$ | 894.6560 | NMR | Heartwood | [9] |
| 1,2-Di-*O*-galloyl-4,6-*O*-(*S*)-hexahydroxydiphenoyl β-D-glucopyranoside | 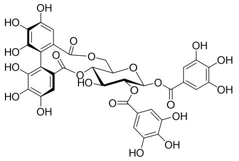 | $C_{34}H_{26}O_{22}$ | 786.5600 | MS | Flower | [10] |

| | | | | | | |
|---|---|---|---|---|---|---|
| | 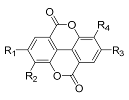 | | | | | |
| Ellagic acid | $R_1 = R_2 = R_3 = R_4 = OH$ | $C_{14}H_6O_8$ | 302.1940 | NMR<br>MS | Peel, flower, leaf | [1, 11, 12] |
| 3,3′-Di-*O*-methylellagic acid | $R_1 = R_3 = OH$, $R_2 = R_4 = OCH_3$ | $C_{16}H_{10}O_8$ | 330.2480 | IR | Seed | [13] |
| 3,3′,4′-Tri-*O*-methylellagic acid | $R_1 = OH$, $R_2 = R_3 = R_4 = OCH_3$ | $C_{17}H_{12}O_8$ | 344.2750 | IR | Seed | [13] |
| 3-*O*-methylellagic acid | $R_2 = OCH_3$, $R_1 = R_3 = R_4 = OH$ | $C_{15}H_8O_8$ | 316.2210 | NMR | Heartwood | [3] |
| 4,4′-Di-*O*-methylellagic acid | $R_1 = R_3 = OCH_3$, $R_2 = R_4 = OH$ | $C_{16}H_{10}O_8$ | 330.2480 | NMR | Heartwood | [3] |
| 3′-*O*-methyl-3,4-methylenedioxy-ellagic acid | $R_1$-$R_2 = OCH_2O$, $R_3 = OH$, $R_4 = OCH_3$ | $C_{16}H_8O_8$ | 328.2320 | NMR | Heartwood | [14] |
| Eschweilenol C<br>(Ellagic acid 4-*O*-α-L-rhamnopyranoside) | 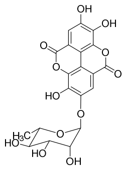 | $C_{20}H_{16}O_{12}$ | 448.3360 | NMR | Heartwood | [14] |
| Ethyl brevifolincarboxylate | 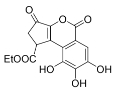 | $C_{15}H_{12}O_8$ | 320.2530 | IR | Flower | [12] |
| | 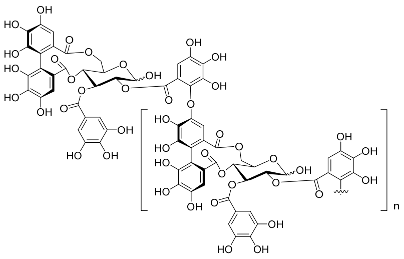 | | | | | |
| Eucalbanin B | n = 1 | $C_{68}H_{50}O_{44}$ | 1571.1040 | NMR | Aril | [15] |
| Eucarpanin $T_1$ | n = 2 | $C_{102}H_{74}O_{66}$ | 2355.6480 | NMR | Aril | [15] |
| Pomegraniin A | n = 3 | $C_{136}H_{98}O_{88}$ | 3140.1920 | NMR | Aril | [15] |
| Pomegraniin B | n = 4 | $C_{170}H_{122}O_{110}$ | 3924.7360 | NMR | Aril | [15] |
| Gallagic acid | 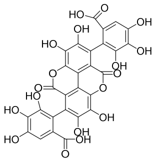 | $C_{28}H_{14}O_{18}$ | 638.4020 | MS | Peel | [11] |
| Gallic acid 3-*O*-β-D-(6′-*O*-galloyl)-glucopyranoside | 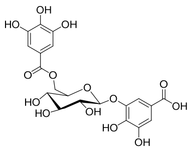 | $C_{20}H_{20}O_{14}$ | 484.3660 | IR | Flower | [2] |

| | | | | | | |
|---|---|---|---|---|---|---|
| 6-*O*-galloyl-2,3-(*S*)-hexahydroxydiphenoyl-D-glucose | 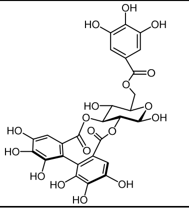 | $C_{27}H_{22}O_{18}$ | 634.4550 | NMR MS | Stem bark, juice | [16, 17] |
| 5-Galloylpunicacortein D | 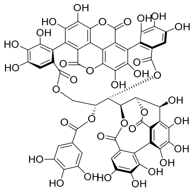 | $C_{55}H_{32}O_{34}$ | 1236.8270 | NMR | Heartwood | [9] |
| 2-*O*-galloylpunicalin (2-*O*-galloyl-4,6-(*S*,*S*)-gallagyl-D-glucose) | 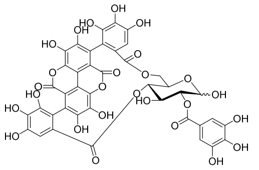 | $C_{41}H_{26}O_{26}$ | 934.6330 | NMR | Heartwood, stem bark | [9, 16] |
| Granatin A | 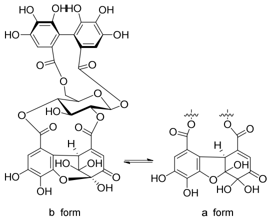 | $C_{34}H_{24}O_{23}$ | 800.5430 | NMR | Fruit, leaf | [7, 18] |
| Granatin B | 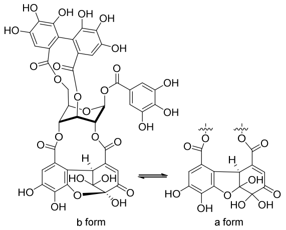 | $C_{41}H_{28}O_{27}$ | 952.6480 | NMR | Fruit, leaf | [7, 18] |
| 2,3-(*S*)-hexahydroxydiphenoyl-D-glucose | 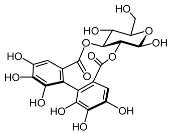 | $C_{20}H_{18}O_{14}$ | 482.3500 | NMR MS | Stem bark, juice | [16, 17] |
| Lagerstannin B | 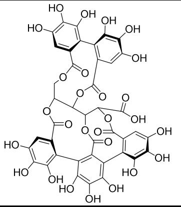 | $C_{41}H_{26}O_{27}$ | 950.6320 | MS | Peel | [19] |
| Lagerstannin C | 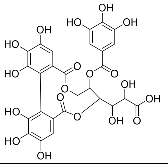 | $C_{27}H_{22}O_{19}$ | 650.4540 | MS | Juice | [17] |

| | | | | | | |
|---|---|---|---|---|---|---|
| 3-*O*-methylellagic acid 4-*O*-α-L-rhamnopyranoside | 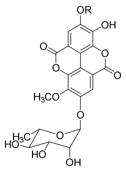 R = H | $C_{21}H_{18}O_{12}$ | 462.3630 | NMR | Heartwood | [3] |
| 3,4'-*O*-dimethylellagic acid 4-*O*-α-L-rhamnopyranoside | R = $CH_3$ | $C_{22}H_{20}O_{12}$ | 476.3900 | NMR | Heartwood | [3] |
| Oenothein B | 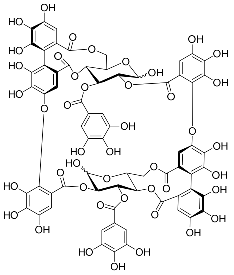 | $C_{68}H_{48}O_{44}$ | 1569.0880 | NMR | Aril | [15] |
| Pedunculagin I | 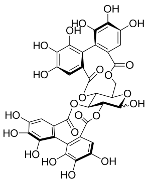 | $C_{34}H_{24}O_{22}$ | 784.5440 | NMR<br>MS | Peel, stem bark | [16, 17] |
| Pedunculagin II | 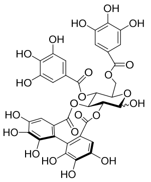 | $C_{34}H_{26}O_{22}$ | 786.5600 | MS | Juice | [17] |
| 1,2,3,4,6-Penta-*O*-galloyl-β-D-glucose | 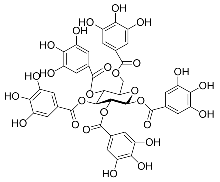 | $C_{41}H_{32}O_{26}$ | 940.6810 | NMR | Leaf | [7] |
| 3,4,8,9,10-Pentahydroxydibenzo[*b*,*d*] pyran-6-one (Urolithin M-5) | 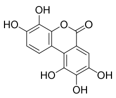 | $C_{13}H_8O_7$ | 276.2000 | NMR | Leaf | [1] |
| Phyllanthusiin E | 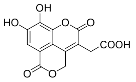 | $C_{13}H_8O_8$ | 292.1990 | NMR | Flower | [12] |
| Pomegranatate | 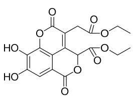 | $C_{18}H_{16}O_{10}$ | 392.3160 | NMR | Flower | [12] |

| | | | | | | |
|---|---|---|---|---|---|---|
| Punicacortein A | 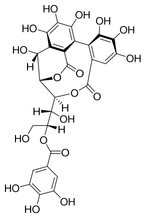 | $C_{27}H_{22}O_{18}$ | 634.4550 | NMR | Stem bark | [5] |
| Punicacortein B | 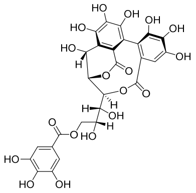 | $C_{27}H_{22}O_{18}$ | 634.4550 | NMR | Stem bark | [5] |
| Punicacortein C<br>Punicacortein D | 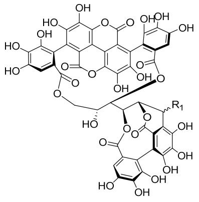<br>$R_1$ = α-OH<br>$R_1$ = β-OH | $C_{48}H_{28}O_{30}$<br>$C_{48}H_{28}O_{30}$ | 1084.7220<br>1084.7220 | NMR<br>NMR | Stem bark, peel<br>Stem bark | [5, 15]<br>[5] |
| Punicafolin | 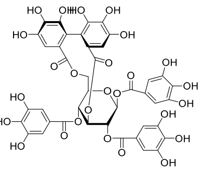 | $C_{41}H_{30}O_{26}$ | 938.6650 | NMR | Leaf | [7] |
| Punicalagin A<br>Punicalagin B | 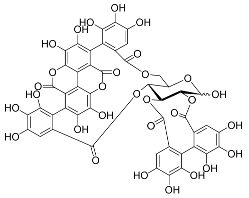<br>α-OH<br>β-OH | $C_{48}H_{28}O_{30}$ | 1084.7220 | NMR<br>MS | Peel, stem bark, aril, juice, root | [11, 15, 16, 19, 20] |
| Punicalin | 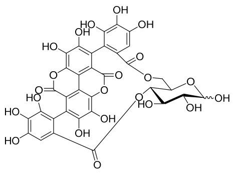 | $C_{34}H_{22}O_{22}$ | 782.5280 | NMR<br>MS | Peel, stem bark, aril, juice, heartwood | [9, 11, 15, 16, 19] |
| Punicatannin A | 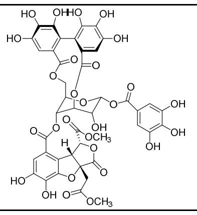 | $C_{43}H_{34}O_{28}$ | 998.7170 | NMR | Flower | [8] |

| | | | | | | |
|---|---|---|---|---|---|---|
| Punicatannin B | 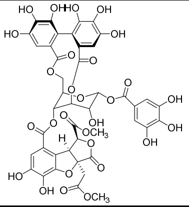 | $C_{43}H_{34}O_{28}$ | 998.7170 | NMR | Flower | [8] |
| Punigluconin | 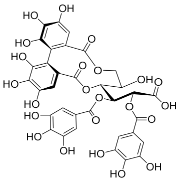 | $C_{34}H_{26}O_{23}$ | 802.5590 | NMR | Stem bark | [5] |
| Strictinin [1-*O*-galloyl-4,6-(*S*) -hexahydroxydiphenoyl-D-glucose] | 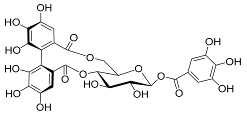 | $C_{27}H_{22}O_{18}$ | 634.4550 | NMR | Leaf | [7] |
| Tellimagrandin I | 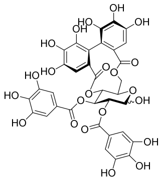 | $C_{34}H_{26}O_{22}$ | 786.5600 | NMR | Peel | [6] |
| Tercatain [1,4-Di-*O*-galloyl-3,6-(*R*)-hexahydroxydiphenoyl-β-glucopyranose] | 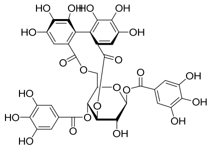 | $C_{34}H_{26}O_{22}$ | 786.5600 | NMR | Leaf | [4] |
| Terminalin (Gallagyl dilactone) | 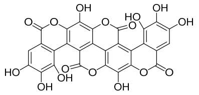 | $C_{28}H_{10}O_{16}$ | 602.3720 | NMR | Stem bark | [16] |
| 1,2,4,6-Tetra-*O*-galloyl-β-D-glucose | 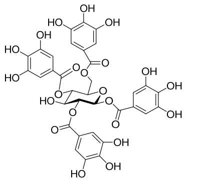 | $C_{34}H_{28}O_{22}$ | 788.5760 | NMR | Leaf | [7] |
| | 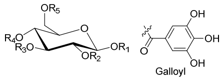 | | | | | |
| 1,2,3-Tri-*O*-galloyl-β-glucopyranose | $R_1 = R_2 = R_3 =$ Galloyl, $R_4 = R_5 =$ H | $C_{27}H_{24}O_{18}$ | 636.4710 | NMR | Leaf | [1] |
| 1,2,4-Tri-*O*-galloyl-β-glucopyranose | $R_1 = R_2 = R_4 =$ Galloyl, $R_3 = R_5 =$ H | $C_{27}H_{24}O_{18}$ | 636.4710 | NMR | Leaf | [4] |
| 1,2,6-Tri-*O*-galloyl-β-glucopyranose | $R_1 = R_2 = R_5 =$ Galloyl, $R_3 = R_4 =$ H | $C_{27}H_{24}O_{18}$ | 636.4710 | NMR MS | Leaf, flower | [1, 10] |
| 1,3,4-Tri-*O*-galloyl-β-glucopyranose | $R_1 = R_3 = R_4 =$ Galloyl, $R_2 = R_5 =$ H | $C_{27}H_{24}O_{18}$ | 636.4710 | NMR | Leaf | [4] |
| 1,4,6-Tri-*O*-galloyl-β-glucopyranose | $R_1 = R_4 = R_5 =$ Galloyl, $R_2 = R_3 =$ H | $C_{27}H_{24}O_{18}$ | 636.4710 | NMR | Leaf | [1] |
| 3,4,6-Tri-*O*-galloyl-β-glucopyranose | $R_3 = R_4 = R_5 =$ Galloyl, $R_1 = R_2 =$ H | $C_{27}H_{24}O_{18}$ | 636.4710 | IR | Flower | [2] |
| Valoneic acid dilactone | 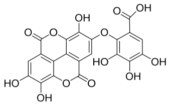 | $C_{21}H_{10}O_{13}$ | 470.2980 | MS | Juice, peel | [19] |

| | | | | | | |
|---|---|---|---|---|---|---|
| **Flavonoids** | | | | | | |
| Hovetrichoside C | 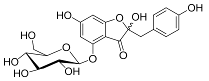 | $C_{21}H_{22}O_{11}$ | 450.3960 | IR | Flower | [2] |
| Phloretin | 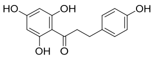 | $C_{15}H_{14}O_5$ | 274.2720 | MS | Juice | [21] |
| Phlorizin | 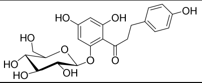 | $C_{21}H_{24}O_{10}$ | 436.4130 | IR | Flower | [2] |
| Eriodictyol-7-*O*-α-L-arabinofuranosyl (1-6)-β-D-glucoside | 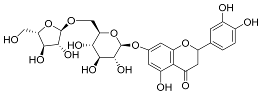 | $C_{26}H_{30}O_{15}$ | 582.5110 | NMR | Stem bark | [22] |
| Granatumflavanyl xyloside | 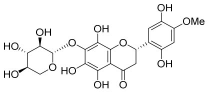 | $C_{21}H_{22}O_{13}$ | 482.3940 | NMR | Flower | [23] |
| Naringin (Naringenin-7-*O*-rhamnoglucoside) | 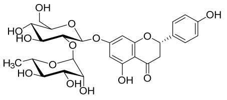 | $C_{27}H_{32}O_{14}$ | 580.5390 | DAD | Peel | [24] |
| Naringenin-4’methyl ether 7-*O*-α-L-arabinofuranosyl(1-6)-β-D-glucoside | 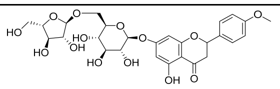 | $C_{27}H_{32}O_{14}$ | 580.5390 | NMR | Stem bark | [22] |
| Pinocembrin | 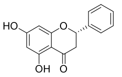 | $C_{15}H_{12}O_4$ | 256.2570 | MS | Juice | [21] |
| Punicaflavanol | 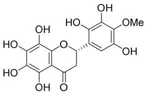 | $C_{16}H_{14}O_{10}$ | 366.2780 | NMR | Flower | [23] |
| Apigenin | 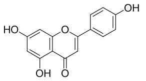 | $C_{15}H_{10}O_5$ | 270.2400 | DAD | Peel | [25] |
| Apigenin 4’-*O*-β-glucopyranoside | 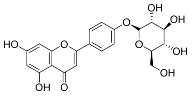 | $C_{21}H_{20}O_{10}$ | 432.3810 | NMR | Leaf | [26] |
| Luteolin | 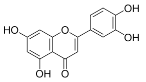 | $C_{15}H_{10}O_6$ | 286.2390 | NMR MS | Peel, flower | [10, 27] |
| Luteolin 3’-*O*-β-glucopyranoside | 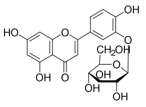 | $C_{21}H_{20}O_{11}$ | 448.3800 | NMR | Leaf | [26] |
| Luteolin 4’-*O*-β-glucopyranoside | 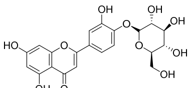 | $C_{21}H_{20}O_{11}$ | 448.3800 | NMR | Leaf | [26] |
| Cynaroside (Luteolin 7-*O*-glycoside) | 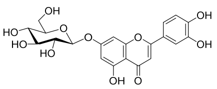 | $C_{21}H_{20}O_{11}$ | 448.3800 | MS | Peel | [28] |
| Luteolin 3’-*O*-β-xylopyranoside | 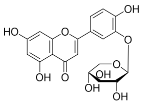 | $C_{20}H_{18}O_{10}$ | 418.3540 | NMR | Leaf | [26] |

| | | | | | | |
|---|---|---|---|---|---|---|
| Tricetin | 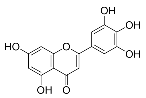 | $C_{15}H_{10}O_7$ | 302.2380 | MS NMR | Flower, peel | [10, 27] |
| Daidzein | 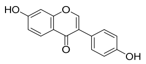 | $C_{15}H_{10}O_4$ | 254.2410 | DAD | Seed | [29] |
| Genistein | 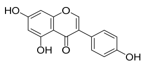 | $C_{15}H_{10}O_5$ | 270.2400 | DAD | Seed | [29] |
| Amurensin (Noricaritin 7-β-D-glucopyranoside) | 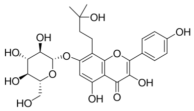 | $C_{26}H_{30}O_{12}$ | 534.5140 | MS | Juice | [30] |
| Kaempferol | 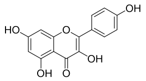 | $C_{15}H_{10}O_6$ | 286.2390 | MS | Peel | [28] |
| Astragalin (Kaempferol 3-*O*-glucoside) | 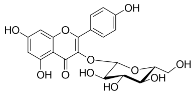 | $C_{21}H_{20}O_{11}$ | 448.3800 | MS | Peel | [31] |
| Kaempferol-3-*O*-rhamnoglucoside | 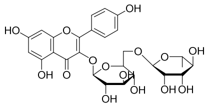 | $C_{21}H_{20}O_{10}$ | 432.3810 | MS | Juice | [21] |
| Myricetin | 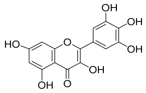 | $C_{15}H_{10}O_8$ | 318.2370 | DAD | Peel | [25] |
| Phellatin | 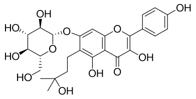 | $C_{26}H_{30}O_{12}$ | 534.5140 | MS | Juice | [30] |
| Quercetin | 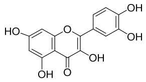 | $C_{15}H_{10}O_7$ | 302.2380 | DAD MS | Juice, leaf, seed, peel | [28, 32] |
| Hirsutrin (Quercetin-3-*O*-glucoside) | 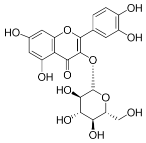 | $C_{21}H_{20}O_{12}$ | 464.3790 | MS | Peel | [31] |
| Quercimeritrin (Quercetin-7-*O*-glucoside) | 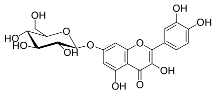 | $C_{21}H_{20}O_{12}$ | 464.3790 | MS | Peel | [31] |
| Quercetin 3-*O*-rhamnoside | 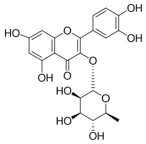 | $C_{21}H_{20}O_{11}$ | 448.3800 | MS | Peel | [31] |
| Rutin (Quercetin-3-*O*-rutinoside) | 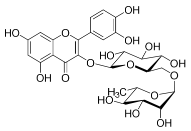 | $C_{27}H_{30}O_{16}$ | 610.5210 | DAD | Juice | [33] |

| | | | | | | |
|---|---|---|---|---|---|---|
| Quercetin-3,4'-dimethyl ether 7-*O*-α-L-arabinofuranosyl(1-6)-β-D-glucoside | 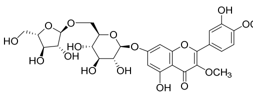 | $C_{28}H_{32}O_{16}$ | 624.5480 | NMR | Stem bark | [34] |
| Cyanidin | 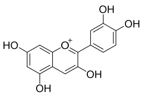 | $C_{15}H_{11}O_6^+$ | 287.2465 | ESR | Juice | [35] |
| Chrysanthemin (Cyanidin-3-*O*-glucoside) | 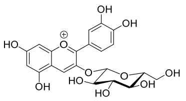 | $C_{21}H_{21}O_{11}^+$ | 449.3875 | MS | Juice | [30] |
| Cyanin (Cyanidin-3,5-di-*O*-glucoside) | 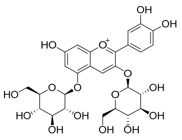 | $C_{27}H_{31}O_{16}^+$ | 611.5285 | MS | Juice | [30] |
| Antirrhinin (Cyanidin-3-*O*-rutinoside) | 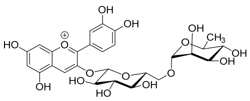 | $C_{27}H_{31}O_{15}^+$ | 595.5295 | MS | Juice | [30] |
| Catechin-cyanidin-3-hexoside | 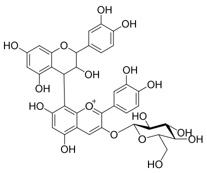 | $C_{36}H_{33}O_{17}^+$ | 737.1715 | MS | Juice | [30] |
| Delphinidin | 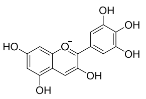 | $C_{15}H_{11}O_7^+$ | 303.2455 | ESR | Juice | [35] |
| Myrtillin (Delphinidin-3-*O*-glucoside) | 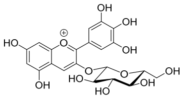 | $C_{21}H_{21}O_{12}^+$ | 465.3865 | MS | Juice | [30] |
| Delphinidin-3,5-di-*O*-glucoside | 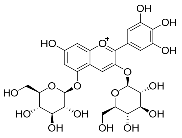 | $C_{27}H_{31}O_{17}^+$ | 627.5275 | MS | Juice | [30] |
| Pelargonidin | 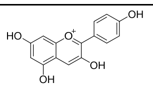 | $C_{15}H_{11}O_5^+$ | 271.2475 | ESR | Juice | [35] |
| Callistephin (Pelargonidin-3-*O*-glucoside) | 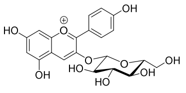 | $C_{21}H_{21}O_{10}^+$ | 433.3885 | MS | Juice | [30] |
| Pelargonin (Pelargonidin-3,5-di-*O*-glucoside) | 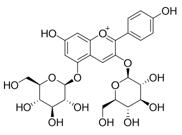 | $C_{27}H_{31}O_{15}^+$ | 595.5295 | MS | Juice | [30] |
| Catechin | 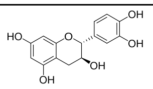 | $C_{15}H_{14}O_6$ | 290.2710 | MS DAD | Peel, juice, leaf | [21, 31, 32] |

| | | | | | | |
|---|---|---|---|---|---|---|
| Epicatechin | 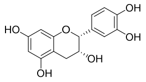 | $C_{15}H_{14}O_6$ | 290.2710 | MS DAD | Peel, juice, leaf, seed | [21, 31, 32] |
| Epicatechin gallate | 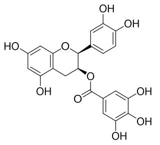 | $C_{22}H_{18}O_{10}$ | 442.3760 | MS | Peel | [31] |
| Epigallocatechin-3-*O*-gallate | 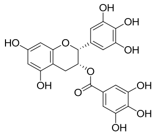 | $C_{22}H_{18}O_{11}$ | 458.3750 | DAD | Fruit | [36] |
| | 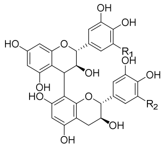 | | | | | |
| Gallocatechin-(4→8)-catechin | $R_1$ = OH, $R_2$ = H | $C_{30}H_{26}O_{13}$ | 594.5250 | MS | Peel | [37] |
| Gallocatechin-(4→8)-gallocatechin | $R_1$ = OH, $R_2$ = OH | $C_{30}H_{26}O_{14}$ | 610.5240 | MS | Peel | [37] |
| Catechin-(4→8)-gallocatechin | $R_1$ = H, $R_2$ = OH | $C_{30}H_{26}O_{13}$ | 594.5250 | MS | Peel | [37] |
| Procyanidin A2 | 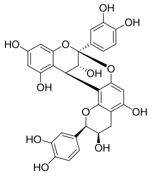 | $C_{30}H_{24}O_{12}$ | 576.5100 | MS | Peel | [31] |
| Procyanidin B1 | 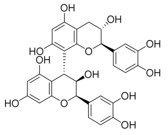 | $C_{30}H_{26}O_{12}$ | 578.5260 | MS | Peel | [31] |
| Procyanidin B2 | 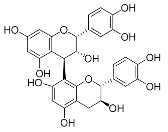 | $C_{30}H_{26}O_{12}$ | 578.5260 | MS | Peel | [31] |
| Procyanidin B3 | 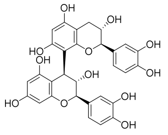 | $C_{30}H_{26}O_{12}$ | 578.5260 | MS | Peel | [31] |
| **Lignans** | | | | | | |
| Conidendrin | 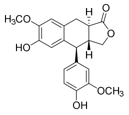 | $C_{20}H_{20}O_6$ | 356.3740 | MS | Juice | [38] |
| Isohydroxymatairesinol | 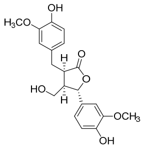 | $C_{20}H_{22}O_7$ | 374.3890 | MS | Peel | [38] |

| | | | | | | |
|---|---|---|---|---|---|---|
| Isolariciresinol | 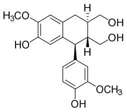 | $C_{20}H_{24}O_6$ | 360.4060 | MS | Juice, peel | [38] |
| Matairesinol | 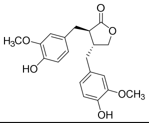 | $C_{20}H_{22}O_6$ | 358.3900 | MS | Wood knot | [39] |
| Medioresinol | 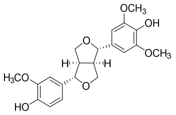 | $C_{21}H_{24}O_7$ | 388.4160 | MS | Juice, wood knot, seed | [39] |
| Phylligenin | 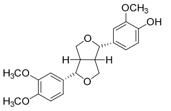 | $C_{21}H_{24}O_6$ | 372.4170 | MS | Peel | [38] |
| Pinoresinol | 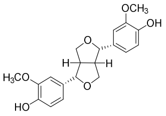 | $C_{20}H_{22}O_6$ | 358.3900 | MS | Juice | [21] |
| Secoisolariciresinol | 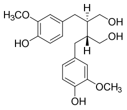 | $C_{20}H_{26}O_6$ | 362.4220 | MS | Peel, juice | [38] |
| Syringaresinol | 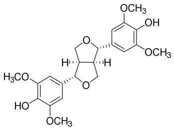 | $C_{22}H_{26}O_8$ | 418.4420 | MS | Juice, wood knot, peel, seed | [39] |
| Pomegralignan | 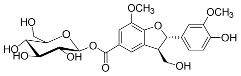 | $C_{24}H_{28}O_{12}$ | 508.4760 | NMR | Aril, peel | [15] |
| Punicatannin C | 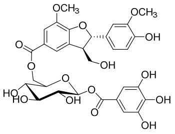 | $C_{31}H_{32}O_{16}$ | 660.5810 | NMR | Flower | [2] |
| **Triterpenoids and phytosterols** | | | | | | |
| Asiatic acid | 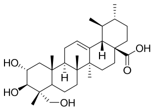 | $C_{30}H_{48}O_5$ | 488.7090 | MS | Flower | [10] |
| Betulinic acid (Betulic acid) | 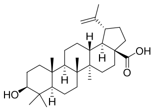 | $C_{30}H_{48}O_3$ | 456.7110 | MP | Leaf | [40] |
| Friedooleanan-3-one (Friedelin) | 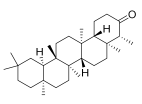 | $C_{30}H_{50}O$ | 426.7290 | IR | Stem and root bark | [41] |
| Maslinic acid | 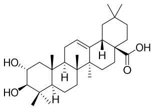 | $C_{30}H_{48}O_4$ | 472.7100 | MS | Flower | [10] |

| | | | | | | |
|---|---|---|---|---|---|---|
| Oleanolic acid | 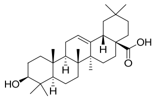 | $C_{30}H_{48}O_3$ | 456.7110 | MS | Flower | [10] |
| Punicanolic acid | 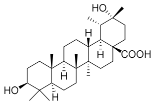 | $C_{30}H_{50}O_4$ | 474.7260 | DAD NMR | Flower, peel | [10, 27] |
| Ursolic acid | 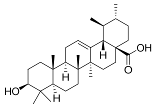 | $C_{30}H_{48}O_3$ | 456.7110 | MS | Flower | [10] |
| Campesterol | 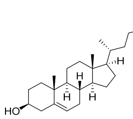 | $C_{28}H_{48}O$ | 400.6910 | MS | Seed | [42] |
| Cholesterol | 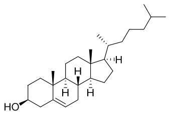 | $C_{27}H_{46}O$ | 386.6640 | MS | Seed | [42] |
| Daucosterol | 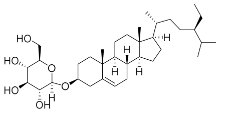 | $C_{35}H_{60}O_6$ | 576.8590 | MS | Seed, flower | [12, 13] |
| β-Sitosterol | 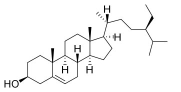 | $C_{29}H_{50}O$ | 414.7180 | MS DAD | Seed, flower | [10, 42] |
| β-Sitosterol laurate | 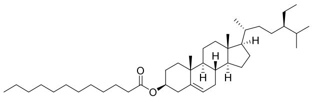 | $C_{41}H_{72}O_2$ | 597.0250 | NMR | Peel | [27] |
| β-Sitosterol myristate | 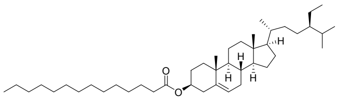 | $C_{43}H_{76}O_2$ | 625.0790 | NMR | Peel | [27] |
| Stigmasterol | 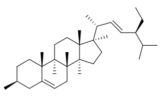 | $C_{29}H_{48}O$ | 412.7020 | MS | Seed | [42] |
| **Alkaloids and indolamines** | | | | | | |
| *N*-(2’,5’-dihydroxyphenyl)pyridinium chloride | 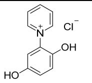 | $C_{11}H_{10}ClNO_2$ | 223.6560 | NMR | Leaf | [26] |
| Hygrine | 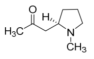 | $C_8H_{15}NO$ | 141.2140 | MS | Root bark | [43] |
| Norhygrine | 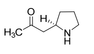 | $C_7H_{13}NO$ | 127.1870 | MS | Root bark | [43] |
| Pelletierine | 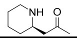 | $C_8H_{15}NO$ | 141.2140 | MS | Stem and root bark | [43] |
| *N*-methylpelletierine | 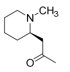 | $C_9H_{17}NO$ | 155.2410 | MS | Stem and root bark | [43] |
| Norpseudopelletierine | 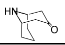 | $C_8H_{13}NO$ | 139.1980 | MS | Stem and root bark | [43] |
| Pseudopelletierine | 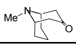 | $C_9H_{15}NO$ | 153.2250 | MS | Stem and root bark | [43] |

| | | | | | | |
|---|---|---|---|---|---|---|
| 2-(2'-Hydroxypropyl)-Δ[1]piperideine | 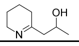 | $C_8H_{15}NO$ | 141.2140 | MS | Root bark | [43] |
| 2-(2'-Propenyl)-Δ[1]piperideine | 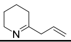 | $C_8H_{13}N$ | 123.1990 | MS | Root bark | [43] |
| Punigratane<br>(2,5-Diheptyl-*N*-methylpyrrolidine) | 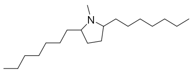 | $C_{19}H_{39}N$ | 281.5280 | NMR | Peel | [44] |
| Sedridine | 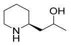 | $C_8H_{17}NO$ | 143.2300 | MS | Root bark | [43] |
| Melatonin | 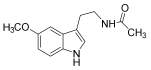 | $C_{13}H_{16}N_2O_2$ | 232.2830 | MS | Fruit extract | [45] |
| Serotonin | 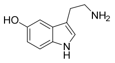 | $C_{10}H_{12}N_2O$ | 176.2190 | FD | Fruit extract | [45] |
| Tryptamine | 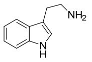 | $C_{10}H_{12}N_2$ | 160.2200 | FD | Fruit extract | [45] |
| **Fatty acids and lipids** | | | | | | |
| Caproic acid<br>(Hexanoic acid) | 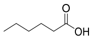 | $C_6H_{12}O_2$ | 116.1600 | MS | Juice | [46] |
| Caprylic acid<br>(Octanoic acid) | 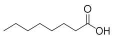 | $C_8H_{16}O_2$ | 144.2140 | MS | Juice | [47] |
| Capric acid<br>(Decanoic acid) | 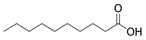 | $C_{10}H_{20}O_2$ | 172.2680 | MS | Juice | [47] |
| Lauric acid<br>(Dodecanoic acid) | 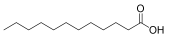 | $C_{12}H_{24}O_2$ | 200.3220 | FID | Seed | [48] |
| Myristic acid<br>(Tetradecanoic acid) | 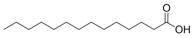 | $C_{14}H_{28}O_2$ | 228.3760 | FID | Seed, fruit | [32] |
| Myristoleic acid<br>(9-*cis*-Tetradecanoic acid) | 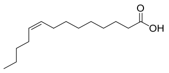 | $C_{14}H_{26}O_2$ | 226.3600 | FID | Seed | [48] |
| Palmitic acid<br>(Hexadecanoic acid) | 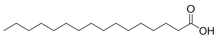 | $C_{16}H_{32}O_2$ | 256.4300 | FID | Seed, fruit | [32, 49] |
| Palmitoleic acid<br>(Hexadec-9-enoic acid) | 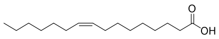 | $C_{16}H_{30}O_2$ | 254.4140 | FID | Seed, fruit | [32] |
| Punicic acid<br>(9*Z*, 11*E*, 13*Z*-octadecatrienoic acid) | 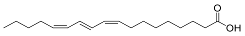 | $C_{18}H_{30}O_2$ | 278.4360 | FID | Seed | [49] |
| Linoleic acid<br>(*cis*, *cis*-9,12-Octadecadienoic acid) | 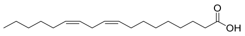 | $C_{18}H_{32}O_2$ | 280.4520 | FID | Seed, fruit | [32, 49] |
| α-Linolenic acid<br>(All-*cis*-9,12,15-octadecatrienoic acid) | 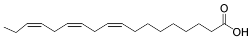 | $C_{18}H_{30}O_2$ | 278.4360 | FID | Seed, fruit | [32] |
| γ-Linolenic acid<br>(All-*cis*-6,9,12-octadecatrienoic acid) | 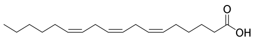 | $C_{18}H_{30}O_2$ | 278.4360 | FID | Seed, fruit | [32] |
| Oleic acid<br>(9*Z*-octadecenoic acid) | 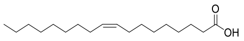 | $C_{18}H_{34}O_2$ | 282.4680 | MS<br>FID | Seed, fruit | [32, 50] |
| Stearic acid<br>(Octadecanoic acid) | 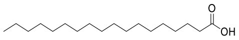 | $C_{18}H_{36}O_2$ | 284.4840 | MS<br>FID | Seed, fruit | [32, 50] |

| | | | | | | |
|---|---|---|---|---|---|---|
| α-Eleostearic acid<br>(9*Z*, 11*E*, 13*E*-octadecatrienoic acid) | 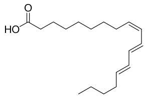 | $C_{18}H_{30}O_2$ | 278.4360 | MS | Seed | [51] |
| β-Eleostearic acid<br>(9*E*, 11*E*, 13*E*-octadecatrienoic acid) | 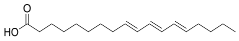 | $C_{18}H_{30}O_2$ | 278.4360 | MS | Seed | [51] |
| Catalpic acid<br>(9*E*, 11*E*, 13*Z*-octadecatrienoic acid) | 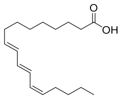 | $C_{18}H_{30}O_2$ | 278.4360 | MS | Seed | [51] |
| Arachidic acid<br>(Eicosanoic acid) | 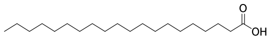 | $C_{20}H_{40}O_2$ | 312.5380 | MS<br>FID | Seed, fruit | [32, 50] |
| Gadoleic acid<br>(9*Z*-icosenoic acid) | 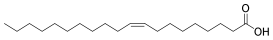 | $C_{20}H_{38}O_2$ | 310.5220 | MS | Seed | [51] |
| Behenic acid<br>(Docosanoic acid) | 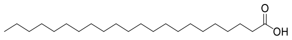 | $C_{22}H_{44}O_2$ | 340.5920 | MS | Seed | [51] |
| Nervonic acid<br>(*cis*-15-Tetracosenoic acid) | 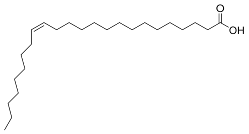 | $C_{24}H_{46}O_2$ | 366.6300 | FID | Seed, fruit | [32] |
| 1-*O*-9*E*,11*Z*,13*E*-octadecatrienoyl glycerol | 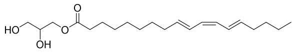 | $C_{21}H_{36}O_4$ | 352.5150 | NMR | Seed, peel | [27, 50] |
| 1-*O*-isopentyl-3-*O*-octadec-2-enoyl glycerol | 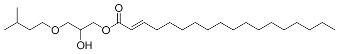 | $C_{26}H_{50}O_4$ | 426.6820 | NMR<br>MS | Seed, peel | [27, 50] |
| Tri-*O*-punicylglycerol | 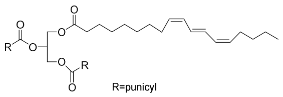 | $C_{57}H_{92}O_6$ | 873.3570 | NMR | Seed | [52] |
| Di-*O*-punicyl-*O*-octadeca-8*Z*, 11*Z*, 13*E*-trienylglycerol | 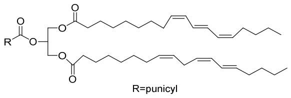 | $C_{57}H_{92}O_6$ | 873.3570 | NMR | Seed | [52] |
| *N*-palmitoyl cerebroside | 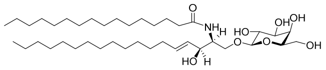 | $C_{40}H_{77}NO_8$ | 700.0550 | TLC<br>FID | Seed | [53] |
| **Organic acids and phenolic acids** | | | | | | |
| Ascorbic acid | 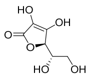 | $C_6H_8O_6$ | 176.1240 | DAD | Leaf, peel, seed, juice | [32] |
| Citric acid | 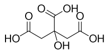 | $C_6H_8O_7$ | 192.1230 | MS<br>DAD | Juice, leaf, peel, seed | [21, 32] |
| Fumaric acid | 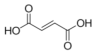 | $C_4H_4O_4$ | 116.0720 | DAD | Juice | [54] |
| L-malic acid | 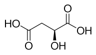 | $C_4H_6O_5$ | 134.0870 | MS<br>DAD | Juice, leaf, peel, seed | [21, 32] |
| Oxalic acid | 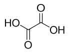 | $C_2H_2O_4$ | 90.0340 | DAD | Juice, leaf, peel, seed | [32, 55] |

| | | | | | | |
|---|---|---|---|---|---|---|
| Quinic acid | 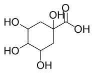 | $C_7H_{12}O_6$ | 192.1670 | DAD | Juice | [33] |
| Succinic acid | 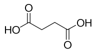 | $C_4H_6O_4$ | 118.0880 | DAD | Juice, leaf, peel, seed | [32, 55] |
| Tartaric acid | 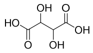 | $C_4H_6O_6$ | 150.0860 | DAD | Juice | [55] |
| Caffeic acid | 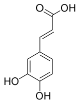 | $C_9H_8O_4$ | 180.1590 | DAD | Peel, juice, seed, leaf | [32, 33] |
| Chlorogenic acid | 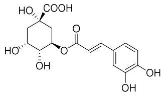 | $C_{16}H_{18}O_9$ | 354.3110 | MS | Juice | [19] |
| Cinnamic acid | 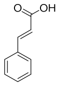 | $C_9H_8O_2$ | 148.1610 | DAD | Juice | [33] |
| *O*-Coumaric acid | 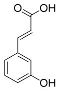 | $C_9H_8O_3$ | 164.1600 | DAD | Juice | [33] |
| *p*-Coumaric acid | 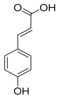 | $C_9H_8O_3$ | 164.1600 | MS DAD | Peel, juice, seed, leaf | [19, 31, 32] |
| *Cis*-*p*-Coumaric acid | 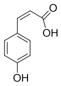 | $C_9H_8O_3$ | 164.1600 | MS | Peel | [31] |
| Coutaric acid | 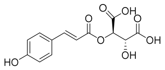 | $C_{13}H_{12}O_8$ | 296.2310 | MS | Peel | [31] |
| 7,8-Dihydroxy-3-carboxymethylcoumarin-5-carboxylic acid | 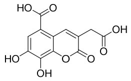 | $C_{12}H_8O_8$ | 280.1880 | NMR | Flower | [2] |
| Ferulic acid | 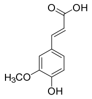 | $C_{10}H_{10}O_4$ | 194.1860 | MS DAD | Juice, seed, peel, leaf | [17, 32] |
| Gallic acid | 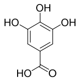 | $C_7H_6O_5$ | 170.1200 | MS | Peel, juice, flower | [17, 19, 23] |
| Methyl gallate | 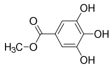 | $C_8H_8O_5$ | 184.1470 | MS | Heartwood | [14] |
| Neochlorogenic acid (5-*O*-caffeoylquinic acid) | 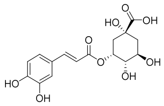 | $C_{16}H_{18}O_9$ | 354.3110 | MS | Peel, juice | [19, 31] |
| Protocatechuic acid | 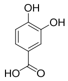 | $C_7H_6O_4$ | 154.1210 | MS | Peel, juice | [19, 31] |

| | | | | | | |
|---|---|---|---|---|---|---|
| Vanillic acid | 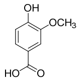 | $C_8H_8O_4$ | 168.1480 | MS | Peel, juice | [17, 31] |
| Coniferyl 9-*O*-[*β*-D-apiofuranosyl(1→6)]-*O*-*β*-D-glucopyranoside | 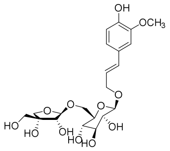 | $C_{21}H_{30}O_{12}$ | 474.4590 | NMR | Seed | [13] |
| Sinapyl 9-*O*-[*β*-D-apiofuranosyl(1→6)]-*O*-*β*-D-glucopyranoside | 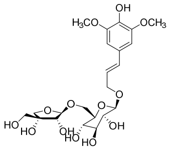 | $C_{22}H_{32}O_{13}$ | 504.4850 | NMR | Seed | [13] |
| **Other compounds** | | | | | | |
| Catechol | 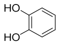 | $C_6H_6O_2$ | 110.1120 | DAD | Juice | [33] |
| Coumestrol | 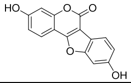 | $C_{15}H_8O_5$ | 268.2240 | DAD | Seed | [29] |
| Icariside D1 | 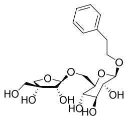 | $C_{19}H_{28}O_{10}$ | 416.4230 | MS | Seed | [13] |
| Phenylethylrutinoside | 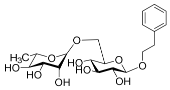 | $C_{20}H_{30}O_{10}$ | 430.4500 | MS | Seed | [13] |
| Syringaldehyde | 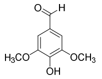 | $C_9H_{10}O_4$ | 182.1750 | MS | Juice | [21] |

## References


1. Nawwar, M. A. M.; Hussein, S. A. M.; Merfort, I., NMR spectral analysis of polyphenols from *Punica granatum*. *Phytochemistry* **1994,** 36, 793-798.
2. Yuan, T.; Wan, C.; Ma, H.; Seeram, N. P., New phenolics from the flowers of *Punica granatum* and their *in vitro* *α*-glucosidase inhibitory activities. *Planta Med* **2013,** 79, 1674-1679.
3. El-Toumy, S.; Rauwald, H., Two new ellagic acid rhamnosides from *Punica granatum* heartwood. *Plant Med* **2003,** 69, 682-684.
4. Hussein, S. A. M.; Barakat, H. H.; Merfort, I.; Nawwar, M. A. M., Tannins from the leaves of *Punica granatum*. *Phytochemistry* **1997,** 45, (4), 819-823.
5. Tanaka, T.; Nonaka, G.-I.; Nishioka, I., Tannins and related compounds. XLI. : Isolation and characterization of novel ellagitannins, punicacorteins A, B, C, and D, and punigluconin from the bark of *Punica granatum* L. *Chem Pharm Bull (Tokyo)* **1986,** 34, 656-663.
6. Satomi, H.; Umemura, K.; Ueno, A.; Hatano, T.; Okuda, T.; Noro, T., Carbonic anhydrase inhibitors from the pericarps of *Punica granatum* L. *Biol Pharm Bull* **1993,** 16, 787-790.
7. Tanaka, T.; Nonaka, G.-I.; Nishioka, I., Punicafolin, an ellagitannin from the leaves of *Punica granatum*. *Phytochemistry* **1985,** 24, 2075-2078.

8. Yuan, T.; Ding, Y.; Wan, C.; Li, L.; Xu, J.; Liu, K.; Slitt, A.; Ferreira, D.; Khan, I. A.; Seeram, N. P., Antidiabetic ellagitannins from pomegranate flowers: inhibition of α-glucosidase and lipogenic gene expression. *Org Lett* **2012,** 14, 5358-5361.
9. El-Toumy, S. A. A.; Rauwald, H. W., Two ellagitannins from *Punica granatum* heartwood. *Phytochemistry* **2002,** 61, 971-974.
10. Xie, Y.; Morikawa, T.; Ninomiya, K.; Imura, K.; Muraoka, O.; Yuan, D.; Yoshikawa, M., Medicinal flowers. XXIII. New taraxastane-type triterpene, punicanolic acid, with tumor necrosis factor-a inhibitory activity from the flowers of *Punica granatum*. *Chem Pharm Bull (Tokyo)* **2008,** 56, 1628-1631.
11. Glazer, I.; Masaphy, S.; Marciano, P.; Bar-Ilan, I.; Holland, D.; Kerem, Z.; Amir, R., Partial identification of bioactive compounds having antifungal activities from *Punica granatum* peel extracts. *J Agric Food Chem* **2012,** 60, 4841-4848.
12. Wang, R.; Wei, W.; Wang, L.; Liu, R.; Yi, D.; Du, L., Constituents of the flowers of *Punica granatum*. *Fitoterapia* **2006,** 77, 534-537.
13. Wang, R.-F.; Xie, W.-D.; Zhang; Xing, D.-M.; Ding, Y.; Wang, W.; Ma, C.; Du, L.-J., Bioactive compounds from the seeds of *Punica granatum* (pomegranate). *J Nat Prod* **2004,** 67, 2096-2098.
14. El-Toumy, S.; Marzouk, M.; Rauwald, H., Ellagi- and gallotannins from *Punica granatum* heartwood. *Pharmazie* **2001,** 56, 823-824.
15. Ito, H.; Li, P.; Koreishi, M.; Nagatomo, A.; Nishida, N.; Yoshida, T., Ellagitannin oligomers and a neolignan from pomegranate arils and their inhibitory effects on the formation of advanced glycation end products. *Food Chem* **2014,** 152, 323-330.
16. Tanaka, T.; Nonaka, G.-I.; Nishioka, I., Tannins and related compounds. XL. : Revision of the structures of punicalin and punicalagin, and isolation and characterization of 2-*O*-galloylpunicalin from the bark of *Punica granatum* L. *Chem Pharm Bull (Tokyo)* **1986,** 34, 650-655.
17. Calani, L.; Beghè, D.; Mena, P.; Del Rio, D.; Bruni, R.; Fabbri, A.; Dall'Asta, C.; Galaverna, G., Ultra-HPLC-MSn (poly)phenolic profiling and chemometric analysis of juices from ancient *Punica granatum* L. cultivars: a nontargeted approach. *J Agric Food Chem* **2013,** 61, 5600-5609.
18. Steinmetz, W. E., NMR assignment and characterization of proton exchange of the ellagitannin granatin B. *Magn Reson Chem* **2010,** 48, 565-570.
19. Fischer, U. A.; Carle, R.; Kammerer, D. R., Identification and quantification of phenolic compounds from pomegranate (*Punica granatum* L.) peel, mesocarp, aril and differently produced juices by HPLC-DAD-ESI/$MS^n$. *Food Chem* **2011,** 127, 807-821.
20. Ono, N.; Bandaranayake, P. C. G.; Tian, L., Establishment of pomegranate (*Punica granatum*) hairy root cultures for genetic interrogation of the hydrolyzable tannin biosynthetic pathway. *Planta* **2012,** 236, 931-941.
21. Mena, P.; Calani, L.; Dall'Asta, C.; Galaverna, G.; García-Viguera, C.; Bruni, R.; Crozier, A.; Del Rio, D., Rapid and comprehensive evaluation of (poly)phenolic compounds in pomegranate (*Punica granatum* L.) juice by UHPLC-$MS^n$. *Molecules* **2012,** 17, 14821-14840.
22. Srivastava, R.; Chauhan, D.; Chauhan, J., Flavonoid diglycosides from *Punica granatum*. *Indian J Chem, Section B* **2001,** 40B, 170-172.
23. Bagri, P.; Ali, M.; Sultana, S.; Aeri, V., New flavonoids from *Punica granatum* flowers. *Chem Nat Compd* **2010,** 46, 201-204.
24. Lansky, E. P.; Newman, R. A., *Punica granatum* (pomegranate) and its potential for prevention and treatment of inflammation and cancer. *J Ethnopharmacol* **2007,** 109, 177-206.

25. Zhao, X.; Yuan, Z.; Fang, Y.; Yin, Y.; Feng, L., Flavonols and flavones changes in pomegranate (*Punica granatum* L.) fruit peel during fruit development. *J Agr Sci Tech* **2014,** 16, 1649-1659.
26. Nawwar, M. A. M.; Hussein, S. A. M.; Merfort, I., Leaf phenolics of *Punica granatum*. *Phytochemistry* **1994,** 37, 1175-1177.
27. Lal, C.; Sharma, M.; Shakyawar, D.; Raja, A.; Sharma, K.; Pareek, P., Natural Dye constituents from rind of *Punica granatum* and its application on Pashmina fabrics. *Arch Appl Sci Res* **2011,** 3, 350-357.
28. van Elswijk, D. A.; Schobel, U. P.; Lansky, E. P.; Irth, H.; van der Greef, J., Rapid dereplication of estrogenic compounds in pomegranate (*Punica granatum*) using on-line biochemical detection coupled to mass spectrometry. *Phytochemistry* **2004,** 65, 233-241.
29. Moneam, N. M. A.; El Sharaky, A. S.; Badreldin, M. M., Oestrogen content of pomegranate seeds. *J Chromatogr* **1988,** 438, 438-442.
30. Gómez-Caravaca, A. M.; Verardo, V.; Toselli, M.; Segura-Carretero, A.; Fernández-Gutiérrez, A.; Caboni, M. F., Determination of the major phenolic compounds in pomegranate juices by HPLC-DAD-ESI-MS. *J Agric Food Chem* **2013,** 61, 5328-5337.
31. Ambigaipalan, P.; de Camargo, A. C.; Shahidi, F., Phenolic compounds of pomegranate byproducts (outer skin, mesocarp, divider membrane) and their antioxidant activities. *J Agric Food Chem* **2016,** 64, 6584-6604.
32. Pande, G.; Akoh, C. C., Antioxidant capacity and lipid characterization of six Georgia-grown pomegranate cultivars. *J Agric Food Chem* **2009,** 57, 9427-9436.
33. Artik, N.; Murakami, H.; Mori, T., Determination of phenolic compounds in pomegranate juice by using HPLC. *Fruit Process* **1998,** 8, 492-499.
34. Chauhan, D.; Chauhan, J. S., Flavonoid diglycoside from *Punica granatum*. *Pharm Biol* **2001,** 39, 155-157.
35. Noda, Y.; Kaneyuki, T.; Mori, A.; Packer, L., Antioxidant activities of pomegranate fruit extract and its Anthocyanidins: delphinidin, cyanidin, and pelargonidin. *J Agric Food Chem* **2002,** 50, 166-171.
36. de Pascual-Teresa, S.; Santos-Buelga, C.; Rivas-Gonzalo, J. C., Quantitative analysis of flavan-3-ols in Spanish foodstuffs and beverages. *J Agric Food Chem* **2000,** 48, 5331-5337.
37. Plumb, G. W.; Pascual-Teresa, S. d.; Santos-Buelga, C.; Rivas-Gonzalo, J. C.; Williamson, G., Antioxidant properties of gallocatechin and prodelphinidins from pomegranate peel. *Redox Rep* **2002,** 7, 41-46.
38. Fischer, U. A.; Jaksch, A. V.; Carle, R.; Kammerer, D. R., Determination of lignans in edible and nonedible parts of pomegranate (*Punica granatum* L.) and products derived therefrom, particularly focusing on the quantitation of isolariciresinol using HPLC-DAD-ESI/MS$^n$. *J Agric Food Chem* **2012,** 60, 283-292.
39. Bonzanini, F.; Bruni, R.; Palla, G.; Serlataite, N.; Caligiani, A., Identification and distribution of lignans in *Punica granatum* L. fruit endocarp, pulp, seeds, wood knots and commercial juices by GC-MS. *Food Chem* **2009,** 117, 745-749.
40. Brieskorn, C.; Keskin, M., Betulic acid in the leaves of *Punica granatum*. *Pharm Acta Helv* **1955,** 30, 361-362.
41. Fayez, M.; Negm, S.; Sharaf, A., Constituents of local plants. V. The constituents of various parts of the pomegranate plant. *Planta Med* **1963,** 11, 439-443.
42. Kaufman, M.; Wiesman, Z., Pomegranate oil analysis with emphasis on MALDI-TOF/MS triacylglycerol fingerprinting. *J Agric Food Chem* **2007,** 55, 10405-10413.
43. Neuhofer, H.; Witte, L.; Gorunovic, M.; Czygan, F., Alkaloids in the bark of *Punica granatum* L. (pomegranate) from Yugoslavia. *Pharmazie* **1993,** 48, 389-391.
44. Rafiq, Z.; Narasimhan, S.; Vennila, R.; Vaidyanathan, R., Punigratane, a novel pyrrolidine alkaloid from *Punica granatum* rind with putative efflux inhibition activity. *Nat Prod Res* **2016,** 25, 1-6.

45. Badria, F., Melatonin, serotonin, and tryptamine in some egyptian food and medicinal plants. *J Med Food* **2004,** 5, 153-157.
46. Mayuoni-kirshinbaum, L.; Tietel, Z.; Porat, R.; Ulrich, D., Identification of aroma-active compounds in 'wonderful' pomegranate fruit using solvent-assisted flavour evaporation and headspace solid-phase micro-extraction methods. *Eur Food Res Technol* **2012,** 235, 277-283.
47. Andreu-Sevilla, A. J.; Mena, P.; Martí, N.; García Viguera, C.; Carbonell-Barrachina, Á. A., Volatile composition and descriptive sensory analysis of pomegranate juice and wine. *Food Res Int* **2013,** 54, 246-254.
48. Akbari, M.; Vaziri, A.; Nasab, A., Comparison of quantitative and qualitative seed oil of pomegranate extracted by cold-press and hexane solvent. *Ind J Fund Appl Life Sci* **2015,** 5, 3704-3709.
49. Fernandes, L.; Pereira, J.; Lopéz-Cortés, I.; Salazar, D.; Ramalhosa, E.; Casal, S., Lipid composition of seed oils of different pomegranate (*Punica granatum* L.) cultivars from Spain. *Int J Food Stud* **2015,** 4, 95-103.
50. Fatope, M. O.; Al Burtomani, S. K. S.; Takeda, Y., Monoacylglycerol from *Punica granatum* seed oil. *J Agric Food Chem* **2002,** 50, 357-360.
51. Topkafa, M.; Kara, H.; Sherazi, S. T. H., Evaluation of the triglyceride composition of pomegranate seed oil by RP-HPLC followed by GC-MS. *J Am Oil Chem Soc* **2015,** 92, 791-800.
52. Yusuph, M.; Mann, J., A triglyceride from *Punica granatum*. *Phytochemistry* **1997,** 44, 1391-1392.
53. Tsuyuki, H.; Ito, S.; Nakatsukasa, Y., Studies on the lipids in pomegranate seeds. *Bull Coll Agric Vet Med Nihon Univ* **1981,** 38, 141-148.
54. Melgarejo, P.; Salazar, D. M.; Artés, F., Organic acids and sugars composition of harvested pomegranate fruits. *Eur Food Res Technol* **2000,** 211, 185-190.
55. Poyrazoğlu, E.; Gökmen, V.; Artık, N., Organic acids and phenolic compounds in pomegranates (*Punica granatum* L.) grown in Turkey. *J Food Com Anal* **2002,** 15, 567-575.


© 2017 by the authors. Submitted for possible open access publication under the terms and conditions of the Creative Commons Attribution (CC BY) license (http://creativecommons.org/licenses/by/4.0/).
